# A rare dominant allele *DYSOC1* determines seed coat color and improves seed oil content in *Brassica napus*

**DOI:** 10.1126/sciadv.ads7620

**Published:** 2025-01-03

**Authors:** Huaixin Li, Mingli Wu, Hongbo Chao, Yongtai Yin, Yutian Xia, Xin Cheng, Kang Chen, Shuxiang Yan, Xiaodong Wang, Yiyi Xiong, Jianjie He, Shipeng Fan, Yiran Ding, Libin Zhang, Haibo Jia, Chunyu Zhang, Maoteng Li

**Affiliations:** ^1^ College of Life Science and Technology, Key Laboratory of Molecular Biophysics of the Ministry of Education, Huazhong University of Science and Technology, Wuhan 430074, China.; ^2^ School of Agricultural Sciences, Zhengzhou University, Zhengzhou 450001, China.; ^3^ Temasek Life Sciences Laboratory, 1 Research Link, National University of Singapore, Singapore 117604, Singapore.; ^4^ Institute of Industrial Crops, Jiangsu Academy of Agricultural Sciences, Key Laboratory of Cotton and Rapeseed, Ministry of Agriculture and Rural Affairs, Nanjing 210014, China.; ^5^ Hubei Bioinformatics and Molecular Imaging Key Laboratory, Wuhan 430074, China.; ^6^ National Key Lab of Crop Genetic Improvement, College of Plant Science and Technology, Huazhong Agricultural University, Wuhan 430070, China.

## Abstract

Yellow seed coat color (SCC) is a valuable trait in *Brassica napus*, which is significantly correlated to high seed oil content (SOC) and low seed lignocellulose content (SLC). However, no dominant yellow SCC genes were identified in *B. napus*. In this study, a dominant yellow SCC *B. napus* N53-2 was verified, and then 58,981 eQTLs and 25 trans-eQTL hotspots were identified in a double haploid population derived from N53-2 and black SCC material Ken-C8. A rare dominant allele *DYSOC1* (*dominant gene of yellow seed coat color and improved seed oil content 1*) was subsequently cloned in a trans-eQTL hotspot that colocated with SCC, SOC, and SLC QTL hotspot on ChrA09 through QTL fine mapping and multi-omics analysis. Transgenic experiments revealed that the expression of *DYSOC1* produced yellow SCC seeds with significantly increased SOC and decreased SLC. Our result provides a rare dominant yellow SCC allele in *B. napus*, which has excellent potential for yellow SCC and high SOC rapeseed breeding.

## Teaser

A rare dominant yellow SCC allele in *B. napus* was identified, which significantly increased SOC and decreased SLC simultaneously.

## INTRODUCTION

Rapeseed (*Brassica napus*, AACC, 2*n* = 38) is one of the most important oilseed crops worldwide, which provides nutritious vegetable oils and proteins for humans and livestock (*1*, *2*). *B. napus* is an allotetraploid that originates from the natural hybridization of *Brassica rapa* (AA, 2*n* = 20) and *Brassica oleracea* (CC, 2*n* = 18) (*1*). In previous studies, it has been concluded that the yellow seed coat color (SCC) seeds always exhibit more excellent properties than black SCC seeds, including a higher seed oil content (SOC), lower seed lignocellulose content (SLC), lower hull content (HC), and lower proanthocyanidin (PA) content (*3*–*6*). Therefore, yellow seeds produce more oil with less pigment and seed meal with less anti-nutrients, which are appreciated by both companies and customers (*7*–*9*). The breeding of yellow SCC *B. napus* varieties has been energetically conducted (*10*–*12*), for example, as a popular cultivar, the yellow SCC variety Yu-yellow No. 1 is cultivated on more than 10 million acres in central China (*7*, *13*). However, *B. napus* varieties with yellow SCC remain relatively rare, and their breeding is still difficult due to the lack of dominant gene resources.

In *Arabidopsis*, yellow SCC results from reduced PA content in the seed coat. In brief, phenylalanine is transformed into *p*-coumaroyl–coenzyme A through the phenylpropanoid biosynthesis pathway, which is further processed for PA synthesis through the flavonoid biosynthesis pathway (*14*). In addition, lignin monomer synthesis nearly exclusively relies on the phenylpropanoid biosynthesis pathway (*15*). Many MYB transcription factors (TFs), along with bHLH-MYB-WD40 (MBW) complexes, zinc finger proteins, and WRKY TFs, have been identified as the primary regulators during this process (*16*–*18*).

The genetic mechanism of yellow SCC is still unclear in *B. napus*, although it has been studied for decades. Most studies have indicated that the SCC in *B. napus* is controlled by at least three pairs of genes, and seeds appear yellow when all genes are homozygous recessive (*19*–*21*). In addition, an incompletely dominant locus that controls yellow SCC has been found in the synthetic line No. 2127-17 (*22*, *23*). It is now widely accepted that yellow SCC in *B. napus* is a complex quantitative trait controlled by multiple loci (*13*, *24*), and the quantitative trait locus (QTL) mapping for SCC, SOC, and SLC has been widely conducted (*13*, *25*–*27*). For instance, Yan *et al.* (*3*) discovered 11, 4, and 12 QTLs for SOC, SCC, and HC, respectively, and one QTL for SOC was tightly linked with the QTLs for HC and SCC on the N8 linkage group (LG). Liu *et al.* (*24*) found a major SCC QTL on ChrA09 in an F_2_ population, which is colocated with major QTLs for SOC and SLC (*5*). The functions of key regulator genes (such as *TTG1*, *TT1*, *TT2*, and *TT8*), structural genes (such as *TT7*, *TT18*, and *TT10*), and transporter genes (*TT12*) of PA biosynthesis have been verified in *B. napus*, the silencing or mutation of which in black SCC materials usually leads to yellow SCC, higher SOC, and lower SLC (*4*, *28*–*30*). However, only a few yellow SCC genes have been identified thus far (*7*, *31*), all of which are recessive, and this recessive nature is inconvenient for yellow SCC rapeseed breeding.

Expression QTL (eQTL) mapping has been extensively used to elucidate the genetic architecture underlying gene expression variation, providing a robust framework for uncovering gene regulatory networks associated with complex quantitative traits (*32*). Integration analysis for QTL and eQTL has become increasingly prominent in the identification of causal genes linked to essential traits, as demonstrated in rice (*33*), soybean (*34*), wheat (*35*), and rapeseed (*36*). QTL fine mapping and map-based cloning are the most widely used strategies for identifying candidate genes within QTLs (*5*, *37*–*39*). For example, the functional gene *GmFATA1B* was cloned through fine mapping from a SOC locus in soybean (*40*). In barley, the amino acid transporter gene *HvBlp*, which controls grain color, was isolated via QTL fine mapping and comparative genomic analysis (*41*).

In our previous studies, QTL mapping for SCC, SOC, and SLC was conducted in the KN double haploid (DH) population derived from parental line N53-2 (which contained yellow SCC loci) and Ken-C8 (which varied considerably in SCC, SOC, and SLC compared with N53-2), and some major QTLs were obtained (*5*, *42*, *43*). In this study, eQTL analysis was conducted to further characterize the genetic mechanism of the SCC, SOC, and SLC variation in the KN DH population. Diversiform variation of eQTL distribution was observed, and a trans-eQTL hotspot overlapping with the major dominant pleiotropy QTL hotspot of SCC, SOC, and SLC was detected on ChrA09. A dominant allele, *DYSOC1* (*dominant gene of yellow seed coat color and improved seed oil content 1*), was subsequently cloned from this trans-eQTL hotspot through QTL fine mapping and map-based cloning, which was verified as a splendid rare locus for yellow SCC, high SOC, and low SLC *B. napus* breeding.

## RESULTS

### Genetic linkage map construction and eQTL identification

The yellow SCC *B. napus* N53-2 was crossed with three black SCC materials [Ken-C8, J2016, and Zhongshuang 11 (ZS11)]. All F_1_ lines produced yellow SCC seeds (fig. S1), indicating a dominant yellow SCC phenotype in N53-2. A DH population was constructed using Ken-C8 and N53-2 as the parents for QTL mapping, and QTLs for SCC and other related traits were identified (*5*, *42*, *43*). In the present study, RNA sequencing (RNA-seq) was conducted on the selfed seeds of DH lines and the parental lines Ken-C8 and N53-2 at 28 days after flowering (DAF), which provided both expression levels of transcribed genes and a comprehensive set of single-nucleotide polymorphisms (SNPs) at the population level. In total, 1299.68-Gb clean data were obtained after eliminating the adaptor sequences and low-quality reads (table S1). After quality trimming, the reads were aligned to the *B. napus* Darmor-*bzh* reference genome, and 756,689 SNPs were detected consequently. The SNP data were further processed for genotyping to identify markers for genetic linkage map construction.

**Fig. 1.**
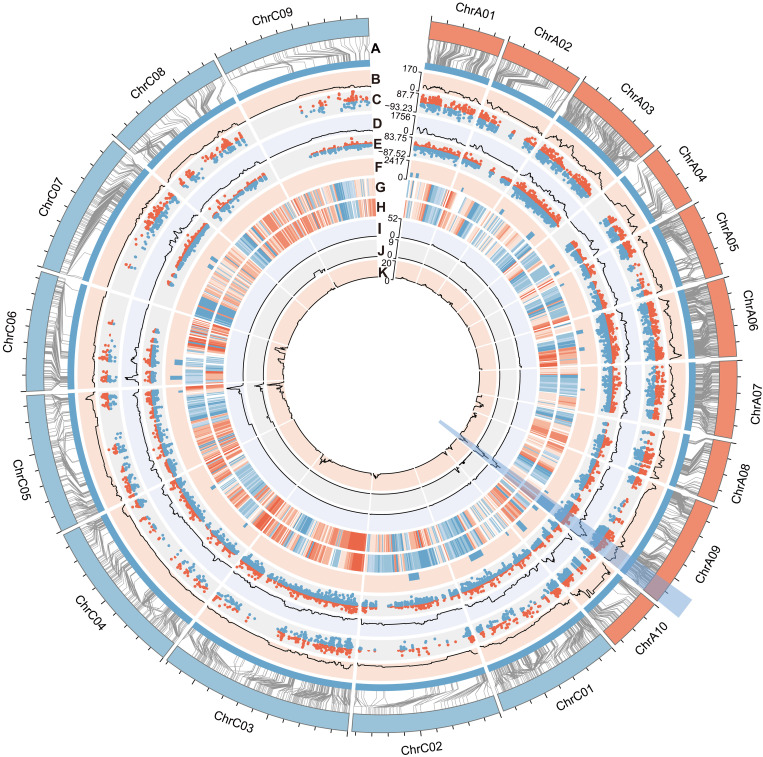
The genetic LG construction and eQTL mapping. (**A**) The collinearity analysis between genetic distance (inner circle) and physical distance (outer circle; the scale represents 5 Mb) of the genetic linkage map. (**B**) The density of cis-eQTLs. (**C**) The *r*^2^ of each cis-eQTL (red and blue points represent the cis-eQTLs with positive and negative additive effects, respectively). (**D**) The density of trans-eQTLs. (**E**) The *r*^2^ of each trans-eQTL (red and blue points represent the trans-eQTLs with positive and negative additive effects, respectively). (**F**) The distribution of the 25 trans-eQTL hotspots (Hotspot 13 was highlighted). (**G** and **H**) The distribution of A and B compartments in Ken-C8 (G) and N53-2 (H) genome (the heatmap was drawn by the compartment value, and the red and blue colors represent A and B compartments, respectively) (*44*). (**I** to **K**) The density of QTLs for SLC (I), SCC (J), and SOC (K) (*5*, *42*, *43*). Among all circles, the QTL and eQTL densities were calculated with a sliding window of 1 Mb and a step size of 100 kb.

After high-quality SNP selection, bin division, and genetic linkage map construction, 3925 SNP markers were successfully assigned to 19 LGs, and a genetic linkage map with a total genetic length of 2933.03 cM was constructed (fig. S2). On average, the distance between the adjacent markers was 0.75 cM, and the map integrity was 99.57%, which ensured the accuracy of genotyping (table S2). Haplotype mapping and collinearity analysis further confirmed the high quality of the newly constructed linkage map (figs. S3 and S4). The fragments per kilobase of exon model per million mapped fragments (FPKM) of each gene was determined as a trait and processed to QTL mapping. In total, 58,981 eQTLs associated with the expression of 31,785 genes (called eGenes) were identified, with an average explained variance (*r*^2^) of 9.95% (Fig. 1 and table S3), including 5811 eQTLs that exhibited an *r*^2^ exceeding 20%. The median genetic distance and physical distance of all eQTLs were 3.13 cM and 697.85 kb, respectively (fig. S5, A and B).

According to the relative distance with corresponding eGenes, 5620 cis-eQTLs and 53,361 trans-eQTLs for 5392 and 29,026 eGenes were identified, respectively (Fig. 1 and fig. S5C). Among the eGenes, 2759, 26,393, and 2633 were regulated by only cis-eQTLs, only trans-eQTLs, and both of them, respectively (fig. S5D). Approximately half of the eGenes were affected by more than one eQTL, indicating a complex regulatory mechanism underlying expression variation in *B. napus* (fig. S6A).

### Characteristics of eQTLs distribution in the genome scale

**Fig. 2.**
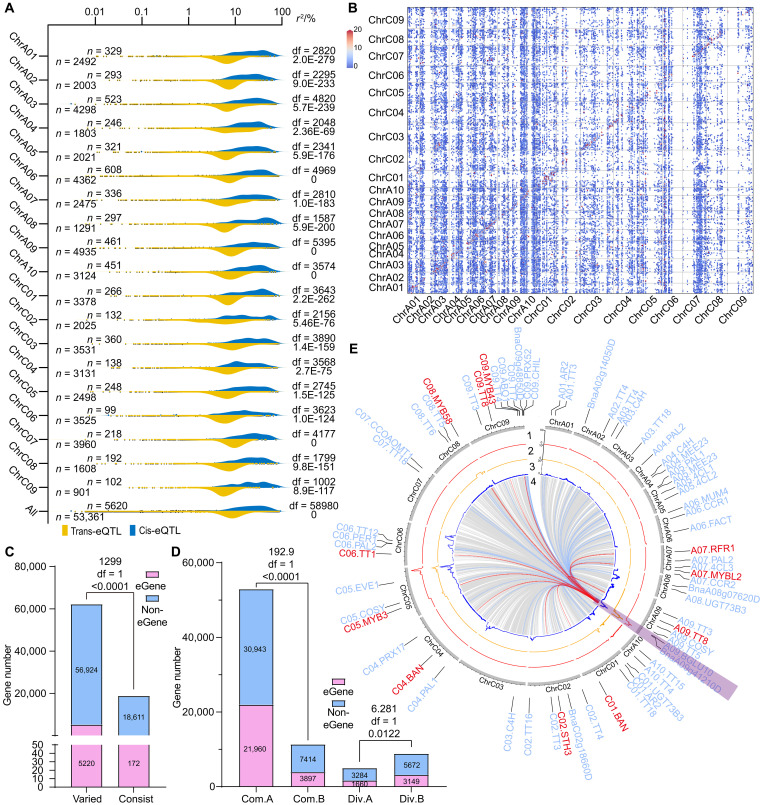
The distribution of eQTLs. (**A**) The *r*^2^ distribution of cis-eQTLs and trans-eQTLs across the 19 chromosomes and the entire genome. Each point represents one eQTL, and the degree of freedoms (df) (above) and *P* values (below) are annotated on the right [two-tailed one-way analysis of variance (ANOVA)]. In addition, the number of cis-eQTL (above) and trans-eQTL (below) are annotated on the left. (**B**) The positions of eQTLs (*x* axis) and their corresponding eGenes (*y* axis) on 19 chromosomes displayed by dot plot. The dot color represents the LOD score of each eQTL. (**C**) The distributions of cis-eQTL eGenes (and non-eGenes) among the genes with sequence variations (“varied”) and without sequence variation (“consist”) between Ken-C8 and N53-2. The chi-square, df, and *P* value are annotated above the histogram (chi-square test). (**D**) The distributions of eGenes (and non-eGenes) among the common (“Com.”) and divergent (“Div.”) A/B compartments of Ken-C8 and N53-2. The chi-square, df, and *P* value are annotated above the histogram (chi-square test). (**E**) The trans-eQTLs within Hotspot 13 (highlighted in purple) and the positions of their corresponding eGenes. The meaning of each circle is as follows: (1 to 3): the QTL density of SLC (1), SCC (2), and SOC (3) (*5*, *42*, *43*); (4) the trans-eQTLs located in Hotspot 13. The blue lines and labels outside represent the structural eGenes of phenylpropanoid and flavonoid biosynthesis pathways, and the red lines and labels represent the regulator genes of those pathways.

The distribution of eQTLs varied considerably across the two sub-genomes and 19 chromosomes. Specifically, 68.77 and 53.98% of the cis- and trans-eQTLs, respectively, were located in the A sub-genome, in which ChrA06 and ChrA09 consisted of the most cis-eQTLs (608) and trans-eQTLs (4935), respectively (fig. S6B). Strong diagonal enrichment was observed on the basis of the relative positions of eGenes and their corresponding eQTLs throughout the genome. In addition, both the LOD score and *r*^2^ of cis-eQTLs were higher than those of trans-eQTLs (Fig. 2, A and B), indicating that cis-eQTLs tend to be more significant and explain more expression variation than the trans-eQTLs.

In our previous studies, genome re-sequencing and Hi-C were conducted on the two parents, Ken-C8 and N53-2 (*42*, *44*). In the present study, 96.81% of the eGenes regulated by cis-eQTLs had sequence variations in the promoter (2 kb before ATG) and/or the coding regions between the two parents. A significant correlation between cis-eQTL and sequence variation was observed (Fig. 2C), indicating that cis-eQTLs were mainly caused by sequence variations within the corresponding eGenes and their promoter regions. The distributions of eGenes in the A and B compartments were also investigated in Ken-C8 and N53-2, revealing significant correlations between eQTLs and both the common and divergent compartments of the parents. Specifically, the eGene frequency of common A compartments (41.51%) was higher than that of common B compartments (34.45%) of the parents. In addition, using N53-2 as the benchmark, the eGene frequency of divergent A compartments (33.58%) was lower than that of divergent B compartments (35.70%) (Fig. 2D).

The correlation between three-dimensional (3D) genome interactions and eQTLs was also evaluated. It was revealed that 6.11 and 13.32% eQTLs were aligned with the common and unique genome interactions of the parents, respectively, while only 2.12 and 6.29% of the randomly created eQTLs (RC.eQTLs) were aligned with the common and unique genome interactions of the parents, respectively, which were significantly lower than those of eQTLs (fig. S5, E and F). Therefore, a significant correlation between eQTLs and 3D genome interactions was detected in the present study.

### A trans-eQTL hotspot that colocated with the QTL hotspot of SOC, SCC, and SLC was identified in ChrA09

A total of 25 trans-eQTL hotspots were identified, each comprising 620 to 2420 trans-eQTLs (Fig. 1 and table S4). Among them, Hotspot 13, located at the end of ChrA09, had the most congregating trans-eQTLs (tables S4 and S5). Further analysis showed that the eGenes regulated by trans-eQTL hotspots were significantly enriched in different Kyoto Encyclopedia of Genes and Genomes (KEGG) pathways and Gene Ontology terms (tables S6 to S30).

Hotspot 13 was colocated with the QTL hotspots of SOC, SCC, and SLC [including neutral detergent fiber content (NDF), acid detergent fiber content (ADF), and acid detergent lignin content (ADL)] (Fig. 2E). In addition, the downstream eGenes of Hotspot 13 were significantly enriched in flavonoid and phenylpropanoid biosynthesis pathways (table S18 and fig. S7A). The trans-eQTLs of a set of enzyme genes of these pathways, such as *C4Hs* (*BnaA03g14010D*, *BnaA04g17570D*, and *BnaC03g16950D*), *PALs* (*BnaA05g07370D*, *BnaC04g08190D*, *BnaA04g04830D*, *BnaA07g16060D*, and *BnaC06g14510D*), and *4CLs* (*BnaA07g25210D*, *BnaA05g19760D*, and *BnaA05g15310D*), as well as regulator genes, such as *TT1* (*BnaC06g08390D*) and *TT8s* (*BnaA09g22810D* and *BnaC09g24870D*), were identified in Hotspot 13 (Fig. 2E). Thus, there should be a pleiotropic gene that represses both the flavonoid and phenylpropanoid biosynthesis pathways and acts as an upstream regulator of SOC, SLC, and SCC in the meantime.

In addition, Hotspot 6 consisted of 779 trans-eQTLs for 772 eGenes that were significantly enriched in flavonoid biosynthesis (table S11 and fig. S7B). The eQTLs of several enzyme genes for flavonoid biosynthesis, such as *TT4* (*BnaC03g06120D*), *CHIs* (*BnaC07g45760D* and *BnaA10g25120D*), and *C4Hs* (*BnaC04g41130D* and *BnaC03g16950D*), were identified within this hotspot (fig. S8A). Two *WD40* genes (*BnaA04g15460D* and *BnaA04g17210D*) were also identified in Hotspot 6 as candidate genes according to previous studies (*16*), and a cis-eQTL of *BnaA04g17210D* was detected (fig. S8C). Hotspot 25 consisted of 1119 trans-eQTLs for 1069 eGenes that were significantly enriched in fatty acid degradation, fatty acid biosynthesis, fatty acid beta-oxidation, and fatty acid catabolic process (table S30 and fig. S7C). Several enzyme genes involved in fatty acid metabolism, including *ECI3* (*BnaC08g09570D*), *ACX3* (*BnaC05g04350D*), *PED1* (*BnaC04g43560D*), and *LACSs* (*BnaC02g28920D* and *BnaA03g29320D*), were identified (fig. S8B). In addition, two *MYB* genes [including *BnaC07g25380D* (*MYB30*) and *BnaC07g27330D* (*MYB78*)] were identified in Hotspot 25 as candidate genes according to previous studies (*45*).

### A pleiotropy allele within Hotspot 13 was identified by the integration of eQTL and QTL fine mapping

**Fig. 3.**
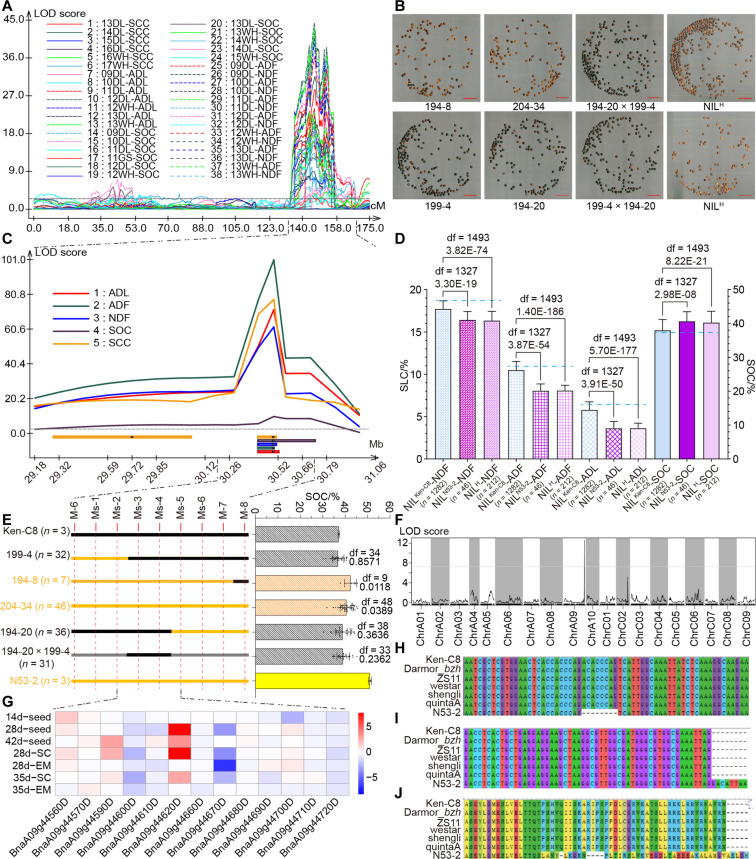
The QTL fine mapping and candidate gene identification of Hotspot 13. (**A**) The QTL mapping of SOC, SCC, NDF, ADF, and ADL on ChrA09 in the KN DH population (*5*, *42*, *43*). (**B**) The matured seeds of BC_5_F_2_ NIL lines. The red bars represent 10 mm. LOD, logarithm of the odds. (**C**) The QTL fine mapping of Hotspot 13 in the BC_5_F_2_ NIL population. (**D**) The SOC (right *y* axis) and SLC (left *y* axis) of different NIL groups. The blue horizontal line denotes the corresponding trait value of Ken-C8. The histograms represent the average values, and the error bars represent the SDs. The df and *P* values are annotated above histograms (two-tailed one-way ANOVA). The number of individuals in each group is annotated behind the *x* axis. (**E**) The genetic background and phenotype of five typical NIL subgroups and their parents. Chromosome fragments inherited from Ken-C8 and N53-2 are marked as black and orange, respectively, and gray indicates heterozygous regions. Names and individual numbers are labeled on the left, and the label colors represent their SCC. The bar graph presents the SOC of those subgroups and their parents, and each point represents one individual. The histograms represent the average values, and the error bars represent SDs. The df and *P* values are annotated above histograms (compared with Ken-C8, two-tailed one-way ANOVA). (**F**) The eQTL mapping of *BnaA09g44670D*. (**G**) Expression profile of the 13 expressed genes within *uqA9-9* in the seed, seed coat (SC), and embryo (EM) of NIL lines. The heatmap was drawn by the log_2_(fold change) of the FPKM of NIL^Ken-C8^ versus NIL^N53-2^. (**H** and **I**) The CDS sequence variation of *BnaA09g44670D* between N53-2 and other *B. napus* materials in the second exon (H) and the terminal region (I). (**J**) The amino acid sequence variation of BnaA09g44670D between N53-2 and other *B. napus* materials.

Because Hotspot 13 was identified as the only trans-eQTL hotspot that colocated with the QTL hotspot of SCC, SOC, and SLC in the KN DH population, the candidate gene within this region needed to be identified. A BC_5_F_2_ near-isogenic line (NIL) population for Hotspot 13 containing 2276 individuals was constructed and QTL fine mapping for SOC, SCC, and SLC was conducted at one time in Wuhan (WH; semi-winter rape-producing area). The QTLs of all those traits were mapped to one highly coincident region that covered a 70-kb interval in the BC_5_F_2_ population, and we named this region *uqA9-9*. It explained 52.02, 37.41, 61.38, 82.88, and 67.18% of the PV of SOC, SCC, NDF, ADF, and ADL, respectively (Fig. 3, A to C and E, and table S31). The BC_5_F_2_ population was further categorized into three groups: the homozygotes that inherited the haplotype of *uqA9-9* from N53-2 (NIL^N53-2^) or Ken-C8 (NIL^Ken-C8^) and the heterozygotes (NIL^H^). The SOC of NIL^N53-2^ was significantly higher than that of NIL^Ken-C8^, while the NDF, ADF, and ADL of NIL^N53-2^ were significantly lower than those of NIL^Ken-C8^ (Fig. 3D). The phenotype of NIL^H^ was similar to that of NIL^N53-2^, while the phenotype of NIL^Ken-C8^ was similar to that of Ken-C8 (Fig. 3D), indicating that the constructed BC_5_F_2_ population had eliminated almost all background interference, and *uqA9-9* contained a dominant allele that could synchronously improve SCC, SOC, and SLC. The same phenomenon was also observed in the winter and spring rape-producing areas, indicating that *uqA9-9* is an environmentally stable locus (fig. S9).

Comparative genomic analysis showed that 17 annotated genes were located in *uqA9-9*, of which 13 were expressed in the NIL seeds. Of the 17 genes, 9 genes had eQTLs, and 3 genes (*BnaA09g44670D*, *BnaA09g44710D*, and *BnaA09g44720D*) had cis-eQTLs (Fig. 3, F and G, and table S32). Meanwhile, the weighted gene co-expression network analysis (WGCNA) was conducted on the basis of the gene expression profiles of all DH lines and the corresponding trait data. A total of 391 and 183 genes that were highly significantly (*P* < 0.01) correlated to SOC and SCC over all inspected microenvironments were detected, with 137 overlapping genes (tables S33 to S35). Among these overlapping genes, *BnaA09g44670D* was the only gene located in *uqA9-9*, which had no homologous genes in *Arabidopsis thaliana* (table S32). Sanger sequencing confirmed that an 8–base pair (bp) Indel existed in the second exon of *BnaA09g44670D* in N53-2, which caused a frameshift mutation (Fig. 3, H to J). Conserved domain prediction indicated that the frameshifted BnaA09g44670D in N53-2 contained a Skp1 domain and was identified as a Skp1-like protein, which is described as the SKP1 components of the Skp1-Cul1-F-box protein (SCF) ubiquitin ligase complex (*46*–*48*). However, no significant domain was observed in the BnaA09g44670D protein of Ken-C8 (fig. S10, A and C). Together, we speculated that the N53-2 allele of *BnaA09g44670D* was the candidate gene within Hotspot 13 and named it *DYSOC1* according to its function.

### Transgenic experiments confirmed that *DYSOC1* was a universal locus for SOC, SCC, and SLC improvement in *B. napus*

**Fig. 4.**
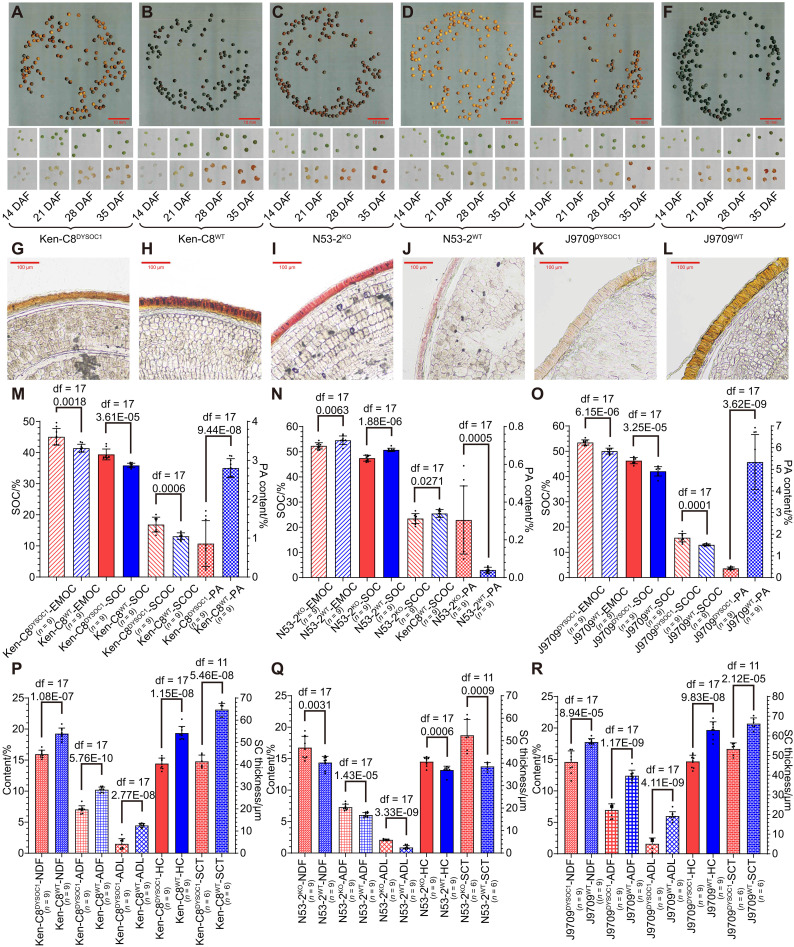
The phenotype of *DYSOC1* transgenic lines. (**A** to **F**) The matured seeds (the first row), intact immature seeds (the second row), and immature seed coats stained by vanillin (the third row) of Ken-C8^DYSOC1^ (A), Ken-C8^WT^ (B), N53-2^KO^ (C), N53-2^WT^ (D), J9709^DYSOC1^ (E), and J9709^WT^ (F). The red bars represent 10 mm. (**G** to **L**) The matured seed slices of Ken-C8^DYSOC1^ (G), Ken-C8^WT^ (H), N53-2^KO^ (I), N53-2^WT^ (J), J9709^DYSOC1^ (K), and J9709^WT^ (L) that stained by phloroglucinol. The red bars represent 100 μm. (**M** to **O**) The EMOC, SCOC, SOC, and PA content of Ken-C8^DYSOC1^ and Ken-C8^WT^ (M), N53-2^KO^ and N53-2^WT^ (N), J9709^DYSOC1^ and J9709^WT^ (O). Among them, EMOC, SOC, and SCOC are plotted on the left *y* axis, and PA content is plotted on the right *y* axis. Each point represents one independent transgenic line, and the histograms represent the average values. The error bars represent SDs, and the df and *P* values are annotated above histograms (two-tailed one-way ANOVA). In addition, the number of independent transgenic lines is annotated behind the *x* axis. Three repetitions were conducted for each line, and the average value was adopted. (**P** to **R**) The NDF, ADF, ADL, HC, and SCT of Ken-C8^DYSOC1^ and Ken-C8^WT^ (P), N53-2^KO^ and N53-2^WT^ (Q), J9709^DYSOC1^ and J9709^WT^ (R). Among them, NDF, ADF, ADL, and HC are plotted on the left *y* axis, and SCT is plotted on the right *y* axis. Each point represents one independent transgenic line, the histograms represent the average values, and the error bars represent SDs, and the df and *P* values are annotated above histograms (two-tailed one-way ANOVA). In addition, the number of independent transgenic lines is annotated behind the *x* axis. Three repetitions were conducted for each line, and the average value was adopted. Specially, the SCT of each material was measured in six randomly selected areas in (G) to (L), respectively.

The full-length genomic sequence of *DYSOC1* (including its promoter) was amplified from N53-2 to construct the complementary vector, which was subsequently transformed into Ken-C8 through *Agrobacterium*-mediated hypocotyl transformation. Compared with wild-type Ken-C8 (Ken-C8^WT^), transgenic lines with *DYSOC1* (Ken-C8^DYSOC1^) represented yellow SCC (Fig. 4, A and B). Further phenotypic investigation revealed that the expression of *DYSOC1* in Ken-C8 led to an 8.80% increase in oil content in the embryo (EMOC), a 28.56% increase in oil content in the seed coat (SCOC), and a 25.37% reduction in HC, lastly resulting in a 9.91% increase in SOC (Fig. 4, M and P). In addition, compared with Ken-C8^WT^, the PA content, seed coat thickness (SCT), NDF, ADF, and ADL in Ken-C8^DYSOC1^ were reduced by 69.30, 36.05, 17.14, 31.05, and 66.12%, respectively (Fig. 4, G, H, M, and P).

A CRISPR-Cas9 gene knockout vector of *DYSOC1* was constructed (fig. S10, A and B) and transformed into N53-2, and the homozygous knockout lines (N53-2^KO^) exhibited darker SCC compared with wild-type N53-2 (N53-2^WT^) (Fig. 4, C and D). Further phenotypic investigation revealed that N53-2^KO^ lines had a 4.07% reduction in EMOC, a 7.90% reduction in SCOC, and a 10.10% increase in HC, which resulted in a 6.49% reduction in SOC (Fig. 4, N and Q). In addition, compared with N53-2^WT^, the PA content, SCT, NDF, ADF, and ADL of N53-2^KO^ were increased by 659.14, 35.84, 16.50, 20.44, and 137.16%, respectively (Fig. 4, I, J, N, and Q).

To evaluate the universality of *DYSOC1*, we transformed the *DYSOC1* complementary vector into J9709, which manifested as black SCC and high SLC. The expression of *DYSOC1* in J9709 (J9709^DYSOC1^) resulted in yellow SCC, a 6.83% increase in EMOC, a 21.79% increase in SCOC, a 25.37% reduction in HC, and a 10.05% increase in SOC (Fig. 4, E, F, O, and R). Compared with wild-type J9709 (J9709^WT^), the PA content, SCT, NDF, ADF, and ADL of J9709^DYSOC1^ were decreased by 92.05, 19.46, 17.83, 43.85, and 73.46%, respectively (Fig. 4, K, L, O, and R). The phenotypic changes mentioned above were consistent with those observed in the Ken-C8 transformation.

**Fig. 5.**
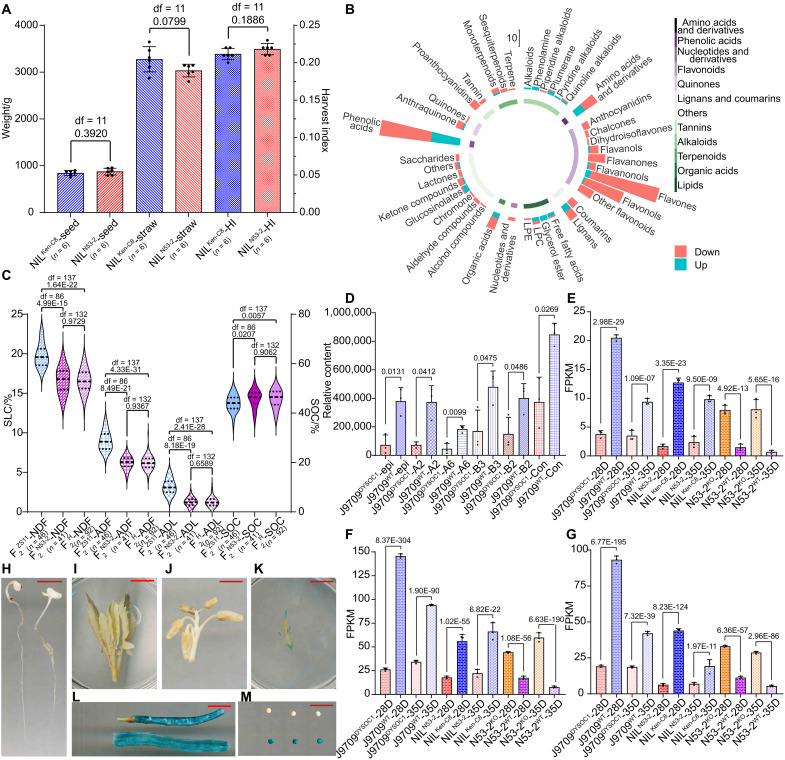
*DYSOC1* restricted PA and lignin monomer synthesis in the seed coat. (**A**) The seed weight (left *y* axis), straw weight (left *y* axis), and harvest index (right *y* axis) of NIL^Ken-C8^ and NIL^N53-2^ plots. Each point represents one plot, and the histograms represent the average values. The error bars represent SDs, and the df and *P* values are annotated above histograms (two-tailed one-way ANOVA). The numbers of plots are annotated behind the *x* axis. (**B**) DAMs detected between the seed coat of J9709^DYSOC1^ and J9709^WT^. The pillar height represents the DAM numbers, with DAM classification annotated in the inner circle. (**C**) The SLC (left *y* axis) and SOC (right *y* axis) of F_2_^ZS11^, F_2_^N53-2^, and F_2_^H^ in the 22WH microenvironment. The top, middle, and bottom horizontal dashed lines of violin plots represent the first, second, and third quartiles of each group, respectively, and the df and *P* values are annotated above violin plots (two-tailed one-way ANOVA). The number of individuals of F_2_^ZS11^, F_2_^N53-2^, and F_2_^H^ are annotated behind the *x* axis. (**D**) The relative content of 4β-8-epicatechin (epi), procyanidin A2 (A2), procyanidin A6 (A6), procyanidin B2 (B2), procyanidin B3 (B3), and coniferaldehyde (Con) in the seed coat of J9709^DYSOC1^ and J9709^WT^. Each point represents one replication, the histograms represent the average values, and the error bars represent SDs. The *P* values are annotated above histograms (Wald test). (**E** to **G**) Expression level of *C06.TT1* (E), *A09.TT8* (F), and *C09.TT8* (G). Each point represents one replication, the histograms represent the average values, and the error bars represent SDs. The *P* values are annotated above histograms (Wald test). (**H** to **M**) The expression profile of *DYSOC1* evaluated by the *GUS* stain in the seedling (H), leaf and stem (I), bud (J), flower (K), pseudoseptum [(L), above], silique peel [(L), below], embryo [(M), above], and seed coat [(M), below]. The red bars represent 10 mm.

Therefore, we confirmed that *DYSOC1* was a universal locus for SOC, SCC, and ADL improvement. In addition, the *DYSOC1* promoter was subsequently amplified for the β-glucuronidase (GUS) stain experiment, demonstrating that *DYSOC1* was highly expressed in the seed coat, silique peel, and pseudo septum. It was slightly expressed in the flower, but no signals were found in nutritive organ, flower bud, or embryo (Fig. 5, H to M).

### *DYSOC1* was promising for high SOC *B. napus* breeding

To evaluate the breeding value of *DYSOC1* in *B. napus*, F_2_ populations were constructed using N53-2 and ZS11, a widely cultivated commercial variety in China that manifested black SCC and medium SLC and shared the same *BnaA09g44670D* DNA sequence as Ken-C8 (Fig. 3, H to J, and fig. S11). The F_2_ populations were cultivated in 2022 and 2023 in Wuhan (abbreviated as 22WH and 23WH, respectively) and genotyped by the *DYSOC1*-specific molecular marker Ms-4 (fig. S12A) and then categorized into three groups: homozygotes containing *DYSOC1* (F_2_^N53-2^), homozygotes lacking *DYSOC1* (F_2_^ZS11^), and heterozygotes (F_2_^H^). The SCC of F_2_^N53-2^ was brighter than that of F_2_^ZS11^, as expected, and the SOC of F_2_^N53-2^ was significantly higher than that of F_2_^ZS11^ with an average increase of 4.04%. The NDF, ADF, and ADL of F_2_^N53-2^ were significantly lower than those of F_2_^ZS11^ by 16.04, 30.98, and 61.96%, respectively, in 22WH (Fig. 5C and fig. S12, B to J). The phenotype of F_2_^H^ was the same as that of F_2_^N53-2^. A similar trend was also observed in 23WH (fig. S13A).

The major FA proportions of the F_2_ population and transgenic lines were also analyzed. Although the content of some FAs significantly changed in the transgenic lines, most of these variances vanished when converted into the FA proportions (figs. S14 to S16). In addition, although the FA composition of ZS11 and N53-2 was diverse, no significant differences were observed in the major FA proportions among different genotypes of the F_2_ population (figs. S11 and S13), as no side effects of *DYSOC1* were found in the major FA composition.

In the transgenic lines, other seed characteristics [seed protein content (SPC) and thousand seed weight] and plant architecture traits (plant height, branch height, main flower length, silique length, seed number per silique, main flower silique number, and branch number) were also investigated, showing no significant side effects of *DYSOC1* (figs. S17 and S18). The plot yields of NIL^Ken-C8^ and NIL^N53-2^ were also measured, revealing no significant differences in seed yield, straw yield, or harvest index (Fig. 5A and fig. S19).

### *DYSOC1* down-regulated the phenylpropanoid and flavonoid biosynthesis pathways and restricted PA and lignin monomer synthesis

To characterize the mechanism of *DYSOC1* controlling SOC, SCC, and SLC, a widely targeted metabolomic analysis was conducted in the mature seed coat of J9709^DYSOC1^ and J9709^WT^. Principal components analysis (PCA) and cluster tree analysis distinguished all replications of J9709^DYSOC1^ and J9709^WT^ (fig. S20, A and D). A total of 255 differential accumulated metabolites (DAMs) were screened from the 1989 detected metabolites (Fig. 5B and fig. S20, B and C). Among them, flavonoids comprised 41.57% of the DAMs (106), with 94.34% (100) being down-regulated in J9709^DYSOC1^. Following flavonoids, phenolic acids account for 18.43% of the DAMs (*47*), with 63.83% (*30*) being down-regulated in J9709^DYSOC1^ (Fig. 5B). These results indicated that the phenylpropanoid and flavonoid biosynthesis pathways were largely down-regulated in the seed coat of J9709^DYSOC1^. In addition, the contents of eight of the 10 detected metabolites directly involved in the lignin monomer biosynthesis were largely decreased in J9709^DYSOC1^, including one DAM (coniferaldehyde). The contents of eight detected PAs were all reduced in J9709^DYSOC1^, including five DAMs (4β-8-epicatechin, procyanidin A2, procyanidin A6, procyanidin B3, and procyanidin B2) (Fig. 5D and fig. S20, E and F), which is consistent with the sharp decline in SLC and PA content in the seed coat of J9709^DYSOC1^.

To investigate the effect of *DYSOC1* at the transcriptional level, transcriptomic analysis was conducted in the 28- and 35-DAF seed coat of J9709^DYSOC1^, J9709^WT^, N53-2^KO^, and N53-2^WT^ (fig. S21). Together with the RNA-seq data of the seed coat of NIL^Ken-C8^ and NIL^N53-2^, almost all KEGG orthologies within phenylpropanoid and flavonoid biosynthesis pathways that participated in PA synthesis were down-regulated by *DYSOC1* (fig. S22). Furthermore, KEGG enrichment analysis of differentially expressed genes (DEGs) of NIL^Ken-C8^ versus NIL^N53-2^, J9709^WT^ versus J9709^DYSOC1^, and N53-2^WT^ versus N53-2^KO^ in the 28- and 35-DAF seed coat revealed that phenylpropanoid and flavonoid biosynthesis pathways were consistently significantly enriched (fig. S23). A total of 26 common down-regulated and 125 common up-regulated DEGs were identified through all six comparisons (fig. S24). Among them, *A9.TT8*, *C9.TT8*, and *C6.TT1* emerged as down-regulated pivotal facilitators of PA synthesis that have been reported before (*29*, *30*, *49*, *50*) (Fig. 5, E to G), which was consistent with the eQTL hotspot analysis (Fig. 2E).

### Evolution and haplotype distribution of *DYSOC1* in *B. napus*

**Fig. 6.**
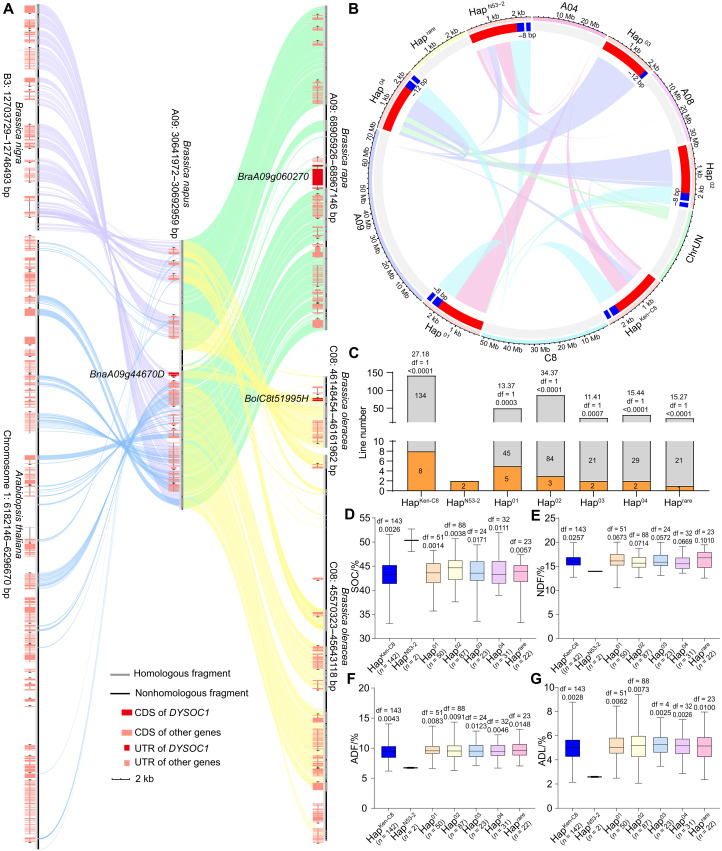
The micro-synteny and haplotype analyses of *DYSOC1*. (**A**) The micro-synteny of *DYSOC1* and its 25-kb flanks with *B. rapa*, *B. nigra*, *B. oleracea*, and *A. thaliana* genome. The query sequence was homologous with the following regions: “A09: 68.91–68.97 Mb,” “B3: 12.70–12.75 Mb,” “C08: 45.57–45.64 Mb and 46.15–46.16 Mb,” and “chromosome 1: 6.18–6.30 Mb” of *B. rapa*, *B. nigra*, *B. oleracea*, and *A. thaliana* genome, respectively. The links indicate the best-aligned position of each query sequence segment relative to the refs. The name of *DYSOC1* and its homology genes are annotated. UTR, untranslated region. (**B**) The variation among six *BnaA09g44670D* haplotypes (Hap^Ken-C8^, Hap^N53-2^, and Hap^01-04^) and their formation through SVs. The links represent the best-aligned position of each haplotype sequence segment relative to the merged AACC genome. The red sections represent promoters, the blue sections represent exons, and the gaps between the blue sections represent introns. The length of Indels in the exons between Hap^Ken-C8^ and other haplotypes is also annotated. (**C**) The yellow SCC frequency of the different *BnaA09g44670D* haplotypes. The frequencies are annotated in the histograms, and the chi-square, df, and *P* value are annotated above the histogram (compared with Hap^N53-2^, chi-square test). (**D** to **G**) The SOC (D), NDF (E), ADF (F), and ADL (G) variations of different *BnaA09g44670D* haplotypes in the *B. napus* natural population. The error bars represent the min and max values of each haplotype, and the top, middle, and bottom parallel lines represent the first, second, and third quartiles of each haplotype, respectively. The df and *P* values are annotated above histograms (compared with Hap^N53-2^, two-tailed one-way ANOVA). In addition, the number of lines of each haplotype is annotated behind the *x* axis. Two replications were cultivated for each line, and the average trait value was adopted.

As previously mentioned, DYSOC1 was identified as a Skp1-like protein. BLAST analysis of *DYSOC1* in Phytozome (https://phytozome-next.jgi.doe.gov/) revealed that the homologous genes were only detected in *B. napus* and its ancestors (*B. rapa* and *B. oleracea*) (*51*). In addition, the sequence identity of DYSOC1 and other known Skp1-like proteins in *A. thaliana* was less than 40% (table S36 and fig. S25), indicating that *DYSOC1* is an orphan gene that evolved within *Brassica*. To further address the evolution of *DYSOC1*, the micro-synteny of *DYSOC1* and its 25-kb flanks were analyzed among *B. napus* (AACC), *B. rapa* (AA), *Brassica nigra* (BB), *B. oleracea* (CC), and *A. thaliana* genomes (Fig. 6A and fig. S26). *A. thaliana* does not have any *DYSOC1* homologous sequence, and *B. nigra* contained only a fragment of the *DYSOC1* coding sequence (CDS), which was not addressed as a gene. *B. rapa* contained the entire *DYSOC1* CDS, while the second exon was interrupted by a 3872-bp insertion that contained a predicted transposable element. *B. oleracea* contained the intact *DYSOC1* CDS, while its promoter was not consistent with that of *B. napus* (Fig. 6A and table S37).

To evaluate the distribution of *DYSOC1* in *B. napus*, a natural population containing 357 lines was collected, and *BnaA09g44670D* and its promoter were amplified in these lines for haplotype analysis. Five main haplotypes (frequency > 5%) were detected, among which one was identical to that of Ken-C8 (named Hap^Ken-C8^), and the other four were named Hap^01^ to Hap^04^. Besides, six rare haplotypes (frequency < 5%) were also isolated, among which one was identical to that of N53-2 (named Hap^N53-2^, which is the *DYSOC1* allele), and the other five were all classified as Hap^rare^ (table S38). Compared with the ancestors *B. rapa* and *B. oleracea*, *BnaA09g44670D* in *B. napus* experienced multiple SVs (chromosome structure variations) across at least four chromosomes, and plentiful haplotypes were consequently formed in the natural population (Fig. 6B). The phenotype of each *B. napus* line in the natural population was measured, which indicated that only Hap^N53-2^ was closely linked with yellow SCC and low SLC. The SOC of Hap^N53-2^ was significantly higher than that of other haplotypes (Fig. 6, C to G). However, no significant differences were observed in other traits between Hap^N53-2^ and other haplotypes (fig. S27).

## DISCUSSION

High SOC, yellow SCC, and low SLC are valuable traits for *B. napus* breeding. Many independent studies have revealed that yellow SCC, high SOC, and low SLC are closely correlated in *B. napus* (*3*, *5*, *52*). Previous studies indicated that yellow SCC is mainly controlled by recessive genes in *B. napus* (*19*–*21*), and all cloned yellow SCC genes are recessive, which limits their application in breeding. For instance, *BnA09MYB47a* promotes flavonoid biosynthesis, and its expression results in black SCC (*7*). Now, only a few dominant loci for yellow SCC have been reported (*22*, *23*), and no dominant genes have been successfully cloned.

Over the last three decades, many QTLs controlling SCC, SOC, SLC, and other related traits have been reported (*3*, *26*, *53*–*56*), with a few being characterized (*7*, *36*, *57*, *58*). For example, *BnaC07.CCRL* was identified as a positive regulator of lignin biosynthesis by combining genome- and transcriptome-wide association studies. Knockdown of *Bna.CCRLs* led to a 4.2% increase in SOC, as well as a 17.6 and 13.1% reduction in SLC and SCT, respectively (*58*). Tan *et al.* (*36*) observed two positive regulators of SOC (*NAC13* and *SCL31*) in *B. napus*, which increased the number of lipid droplets and raised SOC by 10.56 and 8.33%, respectively, in *Arabidopsis*. However, the pleiotropic QTLs are still under characterization, and no genes that simultaneously control SCC, SOC, and SLC have been identified through QTL fine mapping in *B. napus*.

In recent years, the combination of eQTL and QTL mapping has been widely applied to characterize important agronomic traits in crops (*33*–*36*). In this study, eQTL analysis was conducted using a DH population derived from Ken-C8 and N53-2. In total, 58,981 eQTLs for 31,785 eGenes were identified, and approximately half of the eGenes were regulated by more than one eQTL. The cis-eQTLs explained more PV than the trans-eQTLs, consistent with previous studies in *B. napus* and other species (*33*–*36*, *59*). In addition, a significant relationship between eQTLs, sequence variation, and the 3D genome was reported in the present study.

Trans-eQTLs usually affect the expression of eGenes in the form of TFs or certain metabolites. Therefore, many trans-eQTLs cluster together and form eQTL hotspots in certain regions (*60*). In this study, 25 trans-eQTL hotspots were identified genome-wide, which contained key regulators of certain pathways (such as flavonoid biosynthesis and fatty acid degradation pathways). Notably, Hotspot 13, located at the end of ChrA09, regulated the phenylpropanoid and flavonoid biosynthesis pathways and affected SCC, SOC, and SLC pleiotropically (*5*, *42*, *43*). A dominant allele *DYSOC1* was cloned from Hotspot 13 through the integration of eQTL analysis, QTL fine mapping, and map-based cloning. DYSOC1 repressed the expression of *Bn.TT8s* (*BnaA09g22810D* and *BnaC09g24870D*) and *Bn.TT1* (*BnaC06g08390D*) and down-regulated phenylpropanoid and flavonoid biosynthesis pathway genes in the seed coat of *B. napus*, subsequently leading to yellow SCC, low SLC, and thin SCT. Consequently, *DYSOC1* universally improved SOC by up to 10.05%. It is an important advance in the cloning of the dominant yellow SCC gene as well as the pleiotropic gene for SCC, SOC, and SLC in *B. napus*.

Previous studies have demonstrated that PA and lignin synthesis are mainly regulated by TFs, such as MYB, MBW, and WRKY (*16*, *61*). However, regulation through the ubiquitin ligase pathway is rarely reported. Identified as a Skp1-like protein, DYSOC1 is anticipated to form SCF ubiquitin ligase complexes with F-box proteins and CUL proteins (*62*). In these complexes, F-box proteins recognize target proteins and direct them to the CUL1/RBX1 core for ubiquitylation and subsequent degradation (*46*). As far as we know, a few F-box proteins, such as OsFBK1 (*63*), KFB01, KFB20, and KFB50 (*64*), have been identified to mediate the ubiquitylation and degradation of enzymes involved in lignin and PA synthesis. However, the regulatory roles of Skp1-like proteins in these pathways have not yet been investigated. This study presents evidence of Skp1-like protein (DYSOC1) regulating SCC. Therefore, DYSOC1 was supposed to interact with a specific F-box protein, which, in turn, mediates the ubiquitination and degradation of key activating factors involved in the phenylpropanoid and flavonoid biosynthesis pathways.

Overexpressing positive regulators and knocking out negative regulators are possible strategies for improving SOC, SCC, and SLC in *B. napus* (*65*–*70*), but their application in breeding is still challenging. Therefore, searching for naturally dominant alleles is the most effective breeding strategy. As a universally dominant karyogene, *DYSOC1* can be easily induced into any black SCC parent of the crossbreeding through hybridization and then applied to rapeseed crossbreeding (fig. S28). The yellow SCC can serve as a clear phenotypic marker during this process to ensure accuracy. In the present study, the NIL lines and hybridization between N53-2 and ZS11 demonstrated that *DYSOC1* improved SCC, SOC, and SLC in both heterozygotes and homozygotes. More preciously, no unsatisfactory effects were observed on FA composition, plant architecture, or seed yield due to *DYSOC1*. The rarity of Hap^N53-2^ in the natural *B. napus* population (2 of the 357 lines) further highlights the potential of *DYSOC1* allele in *B. napus* breeding. Although some researchers expressed concerns about the shortcomings of yellow SCC seeds (*71*, *72*), these challenges are expected to be addressable through suitable breeding strategies and locus selection (fig. S28). On the basis of the existing results, the widespread application of *DYSOC1* could substantially increase rapeseed oil production.

## MATERIALS AND METHODS

### Plant materials and field experiment

The KN DH population and its parental lines, Ken-C8 and N53-2, were first described by Wang *et al.* (*73*). Ken-C8, the male parent of the KN DH population, is a spring *B. napus* manifested low SOC (around 40%), high SLC, and black SCC. In contrast, N53-2, the female parent of the KN DH population, is a winter *B. napus* manifested high SOC (over 50%), low SLC, and yellow SCC. Besides, ZS11 is a widely cultivated commercial variety in China, and J9709 is a widely used spring *B. napus* line in transgenic research.

In this study, all plant materials were cultivated in the field under natural conditions in Wuhan, Hubei Province (semi-winter rape-producing area, 113.68°E, 30.58°N) unless otherwise stated. The KN DH population was cultivated in Yangling (winter rape-producing area, 108.08°E, 34.27°N) in 2019 for eQTL sample collection. The BC_5_F_2_ NIL lines were cultivated in Wuhan, Yangling, and Xining (spring rape-producing area, 101.49°, N36.34°). The field management adhered to the regular agricultural requirements described by Chao *et al.* (*42*).

### Sample collection and the RNA-seq of the KN DH population

The 28-DAF selfed seeds were collected from 156 different DH lines and the parental lines Ken-C8 and N53-2 for RNA extraction, library construction, and RNA-seq analysis following the reported procedures (*74*). For quality control, all raw reads were filtered to remove low-quality reads, adapter sequences, and reads with *Q* < 30 bases. The clean data of tall samples were mapped to the *B. napus* Darmor-*bzh* reference genome using STAR software, and these mapped reads were used for FPKM calculation and subsequent analysis.

### SNP calling and genotyping

SNP loci were identified and selected by Genome Analysis Toolkit (GATK) software (*75*), and the low-quality SNPs were filtered out. The retained SNP markers were used for genotyping to facilitate subsequent genetic analysis.

The genotype encoding followed the two alleles encoding rules, which were commonly used in genetic research (*74*). All polymorphism markers were coded into eight segregating patterns (ab × cd, ef × eg, hk × hk, lm × ll, nn × np, aa × bb, ab × cc, and cc × ab), and the markers with the segregation pattern of aa × bb were selected to match with the DH population used in this study. After SNP selection and bin division, a total of 4005 effective SNP markers were selected for the linkage analysis.

### Linkage map construction, evaluation, and eQTL analysis

HighMap software was used to arrange the SNPs, and the genetic distances among SNPs were calculated using the Kosambi mapping function (*76*). In total, 3925 of the 4005 effective SNP markers were assigned to 19 LGs. The maximum likelihood method, SMOOTH algorithm, and Detaily MSTmap were used to construct the genetic linkage map and correct genotyping errors.

The Composition Internal Mapping (CIM) method and the RQTL software were used for eQTLs mapping. The 1000 permutation tests were performed to determine the thresholds of eQTLs. According to the relative distance between eQTLs and the corresponding eGenes, the eQTLs were further classified as cis-eQTLs (<1 Mb) or trans-eQTLs (>1 Mb or located on different chromosomes). The eQTL density was calculated with a sliding window of 1-Mb window and a step size of 100 kb, and the 95% value served as the threshold for trans-eQTL hotspot identification.

### Weighted gene co-expression network analysis

The SOC and SCC of the KN DH population in different microenvironments have been published before (*5*, *42*). In the present study, the co-expression network analysis was performed using the R package WGCNA v1.7.3. Specifically, the “adjacency” function in WGCNA was used to calculate the adjacencies with a soft-thresholding power of seven. To identify modules of co-expressed genes, the function “TOMsimilarity” was used to calculate the topological overlap matrix (TOM) between all genes. Then, the TOM values were used as input for average linkage hierarchical clustering. The clustered gene trees were cut into different modules using the dynamic shearing method. The module eigengene (ME) was summarized for each module. Last, association analyses were conducted between merged ME and phenotypic traits (*77*).

### RC.eQTL generation

The distribution of eQTL length and the number of eQTLs for each eGenes were first analyzed. Then, a series of genomic regions were randomly generated across the 19 chromosomes and matched with 1000 randomly selected genes on the basis of the observed eQTL length and number distribution. These randomly selected genes and corresponding randomly generated genomic regions were regarded as the RC.eQTLs.

### Trait investigation

For NIL and the F_2_ populations of ZS11 × N53-2, the matured seeds of each individual were collected for seed traits analysis. As for the *B. napus* natural population, transgenic lines, and their corresponding WT lines, each line was cultivated for two rows. For each line, the matured seeds from five healthy individuals were pooled for seed traits analysis. In addition, the *B. napus* natural population was cultivated for two replications.

In the present study, the SOC, SLC, SPC, and FA composition of the NIL lines, F_2_ populations of ZS11 × N53-2, and the *B. napus* natural population were measured by the NIRS method using NIRS Systems 5000 (Foss, Denmark) (*78*–*80*). The SPC and SLC of the transgenic lines and their corresponding WT lines were also analyzed by NIRS, while their SOC and FA composition were measured by gas chromatography (GC) as previously described (*81*). Total FAs were extracted using 2.5% H_2_SO_4_-methanol, with heptadecanoic acid as the internal reference. Major FA content was measured using the 7890A chromatographic column (Agilent, USA) in the gas chromatography system (Agilent, USA) following the instructions. Three repetitions were conducted for each measurement, and the average values were adopted. The SOC, SLC, SPC, HC, PA content, and FA content described in the present study represent the mass ratio of each component relative to the total seed weight.

### SCT and lignin content analysis of seed coat via hard-tissue slicing and phloroglucinol stain

The mature seeds were fixed in 70% Formalin-Aceto-Alcohol (FAA) solution for 2 days, followed by dehydration, methyl methacrylate (MMA) infiltration, and MMA embedding. The seeds were sectioned into 10-μm slices in the middle and placed in a 60°C oven overnight. Slices were stained with phloroglucinol staining solution (Servicebio, China) for 30 s after rehydration and mordanting. The excess staining solution was removed, and the images were captured within 5 min.

### PA content analysis in the seed coat of *B. napus*

The PA of mature *B. napus* seed coats was extracted using 2.5% butanol-HCl (*82*). Meanwhile, for the standard curve, the PA standard (Macklin, Shanghai, China) was dissolved in methanol and extracted following the same protocol. The absorbance of the mixture was measured at 545 nm using a FlexStation 3 multimode reader (Molecular Devices, USA) (figs. S29 and S30). The PA concentration of the extracted seed coat solutions was calculated on the basis of the standard curve.

Vanillin turns red upon binding to PA. The 1% vanillin staining solution was prepared as reported before (*72*) and balanced with 12 mol/L HCl before use. The immature seed coats were soaked in the staining solution for 10 min at room temperature before images were captured.

### NIL construction and QTL fine mapping

The genetic background of each individual of the KN DH population was screened, and two lines (I-094 and I-179) that inherited the superior genotype of Hotspot 13 from N53-2 were screened out, which were continuously backcrossed with Ken-C8 five times to construct the BC_5_ population. Subsequently, the BC_5_F_2_ population was constructed by selfing the BC_5_ individuals. In addition, to increase the genetic diversity of the BC_5_F_2_ population and to improve the accuracy of QTL fine mapping, two BC_5_ individuals (194-20 and 199-4) with interchanged chromosomes within Hotspot 13 and exhibited black SCC were crossed, and their hybrids were also used for BC_5_F_2_ population construction.

Meanwhile, the Indels within Hotspot 13 between the genomes of Ken-C8 and N53-2 were screened out to develop codominant molecular markers, as reported before (*44*). Six molecular markers were selected for QTL fine mapping (table S39). The fine mapping of Hotspot 13 in the BC_5_F_2_ population was subsequently processed using WinQTLCart2.5 software and the CIM method, as described before (*42*).

### Vector construction and the transformation of *B. napus*

The unabridged sequence of *DYSOC1*, including its native promoter, was amplified from the genomic DNA of N53-2 and cloned into the pCAMBIA1303 plasmid to construct the complementary vector. To guarantee that *DYSOC1* was driven by its native promoter, the *CaMV35S* promoter in the pCAMBIA1303 plasmid was eliminated. The CRISPR-Cas9 target site was designed using the online tool (http://crispr.hzau.edu.cn/CRISPR2/) (*83*), and the gene knockout vector was constructed using pHSN401 plasmid as the backbone.

The complementary vector and CRISPR-Cas9 gene knockout vector were transformed into *B. napus* via *Agrobacterium*-mediated hypocotyl transformation (*81*). In consideration of the hygromycin B resistance gene in those vectors, the empty pCAMBIA1303 and pHSN401 plasmid were also transformed into *B. napus*, which were referred to as wild-type in this study for unambiguous demonstration.

### Identification of positively transformed plantlets and CRISPR-Cas9 on-target mutants in *B. napus*

The genomic DNA of each transgenic *B. napus* individual was extracted using the NuClean PlantGen DNA Kit (ComWin, Beijing, China) according to the manual. Vector-specific primers were designed to identify positive transgenic plantlets via polymerase chain reaction. As for *DYSOC1* gene knockout plantlets, the CRISPR-Cas9 target site of *DYSOC1* was amplified for Sanger sequencing, and the on-target mutations were screened. Nine lines with different *DYSOC1* on-target mutations were identified (fig. S10B), and nine independent positively transformed plantlets were generated for each complementary vector transgenic experiment. The primers mentioned above have been listed in table S39. Those transgenic lines were subcultured to the T3 generation for trait measurement.

### The expression pattern analysis of *DYSOC1*

The promoter of *DYSOC1* was amplified from the genomic DNA of N53-2 and cloned into the pCAMBIA1305 plasmid with the *GUS* gene on the heels, and the *CaMV35S* promoter was removed beforehand. The *DYSOC1* promoter reconstituted GUS vector was transformed into J9709 via *Agrobacterium*-mediated hypocotyl transformation. Positively transformed planta was screened out as described above and subcultured to T3 generation. Tissues from these plants were collected and stained with the GUS Stain Kit (Coolaber, Beijing, China) overnight under room temperature following the instruction. Images were captured after decolorizing with absolute ethanol.

### RNA-seq analysis of NIL and transgenic materials

For seed coat and embryo samples, the seed coat and embryo were separated and immediately soaked in the RNA storage solution (Invitrogen, USA). The RNA-seq samples were named after “line name-stage-tissue-replication”; in addition, “seed coat,” “embryo,” and “DAF” were abbreviated as “SC,” “EM,” and “d,” respectively. For example, “NIL^N53-2^-35d-SC-1” represents the first replication of the 35-DAF seed coat of NIL^N53-2^ (tables S40 and S41).

Total RNA extraction, library construction, and sequencing were performed as described before. The clean reads were mapped to Darmor_*bzh B. napus* reference genome, and DEGs were identified using the Deseq2 R package with |log_2_(fold change)| > 1, *P* value < 0.05 and *P* adj. value < 0.05 in the meantime. The comparisons between different samples were presented as “A versus B,” indicating the relative expression level of genes in sample “B” compared with that in sample “A.”

### Widely targeted metabolomic analysis processed by UPLC-MS/MS

Three independent lines of J9709^WT^ and J9709^DYSOC1^ were randomly selected, and the seed coats of their mature seeds were collected for metabolomics analysis. All samples were frozen with liquid nitrogen, vacuum freeze dried using a Scientz-100F Lyophilizer (Xinzhi, China), and ground into powder. The sample powder was extracted with 70% methanol solution and filtered with 0.22-μm microfiltration membrane before ultra-performance liquid chromatography (UPLC) (ExionLC AD, Sciex, USA) and tandem mass spectrometry (MS/MS) (Applied Biosystems 4500 QTRAP, USA). The detailed parameters of UPLC and MS/MS followed the reported protocol (*84*). The DAMs were selected with |log_2_(fold change)| > 1 and *P* value < 0.05.

### Statistical analysis

The two-tailed one-way analysis of variance (ANOVA) assessed the significance of trait variation between different materials or genotypes. The chi-square test was used to evaluate the significance of the correlation between eQTL and gene sequence variation. The chi-square test was used to assess the significance of the correlation between yellow SCC and different *DYSOC1* haplotypes. The significant level was classified as follows: *P* ≤ 0.05 (significant, “*”), *P* value ≤ 0.01 (highly significant, “**”), and *P* > 0.05 (nonsignificant, “n.s.”).

### The evolution analysis of *DYSOC1*

The conserved domain and tertiary structure of *DYSOC1* were predicted using the Conserved Domain Database (www.ncbi.nlm.nih.gov/cdd) and AlphaFold2 (https://colab.research.google.com/github/sokrypton/ColabFold/blob/main/AlphaFold2.ipynb) websites, respectively. Homologous sequences of *DYSOC1* and the 25-kb flanks were searched in the *B. rapa* (Chiifu.v4), *B. nigra* (Ni100 LR v2.0), *B. oleracea* (HDEM.v0), and *A. thaliana* (TAIR10) genome using BLAST (https://ftp.ncbi.nlm.nih.gov/blast/executables/blast+/) with default parameters. The *B. rapa*, *B. nigra*, and *B. oleracea* genomes were downloaded from BnIR (https://yanglab.hzau.edu.cn/BnIR) (*85*), and the *A. thaliana* genome was downloaded from TAIR (www.arabidopsis.org/) (*86*). The syntenic region of the entire query in each ref genome was first determined, and only the best-aligned sequence of each query segment within the syntenic region was retained for the micro-synteny presentation. In addition, the unabridged BLAST output was presented in fig. S26. The sequence of each *BnaA09g44670D* haplotype was blasted against the merged AACC genome (merged *B. rapa* and *B. oleracea* genome), and the best-aligned sequence of each query segment relative to the ref genome was retained to evaluate the origin of each *BnaA09g44670D* haplotype.

The homology between DYSOC1 and the *A. thaliana* Skp1-like proteins was calculated using the BLAST. The phylogenic tree was constructed using MEGA 10 software and the maximum likelihood method under default parameters (*87*).
