# Supplementary material for: A rare dominant allele DYSOC1 determines seed coat color and improves seed oil content in Brassica napus
Source: Sci Adv. 2025 Jan 3;11(1):eads7620. doi: 10.1126/sciadv.ads7620 (PMC11698099; doi:10.1126/sciadv.ads7620)
Supplement: Supplementary file 1 — Supplementary Text Figs. S1 to S30 Legends for tables S1 to S41 [file sciadv.ads7620_sm.pdf]

Science Advances
AAAS

Supplementary Materials for

A rare dominant allele *DYSOC1* determines seed coat color and improves seed oil content in Brassica napus

Huaixin Li *et al.*

Corresponding author: Maoteng Li, limaoteng426@hust.edu.cn; Chunyu Zhang, zhchy@mail.hzau.edu.cn

Sci. Adv. **11**, eads7620 (2025)
DOI: 10.1126/sciadv.ads7620

The PDF file includes:

Supplementary Text
Figs. S1 to S30
Legends for tables S1 to S41

Other Supplementary Material for this manuscript includes the following:

Tables S1 to S41

Supplementary Text

The supplementary figures that could support the data quality and conclusions of the main paper are as follows. Which were numbered in the order of their citation in the main text. The supplementary tables are too big to fit this Word file, which was provided in the other separate file (Data S1.xlsx).

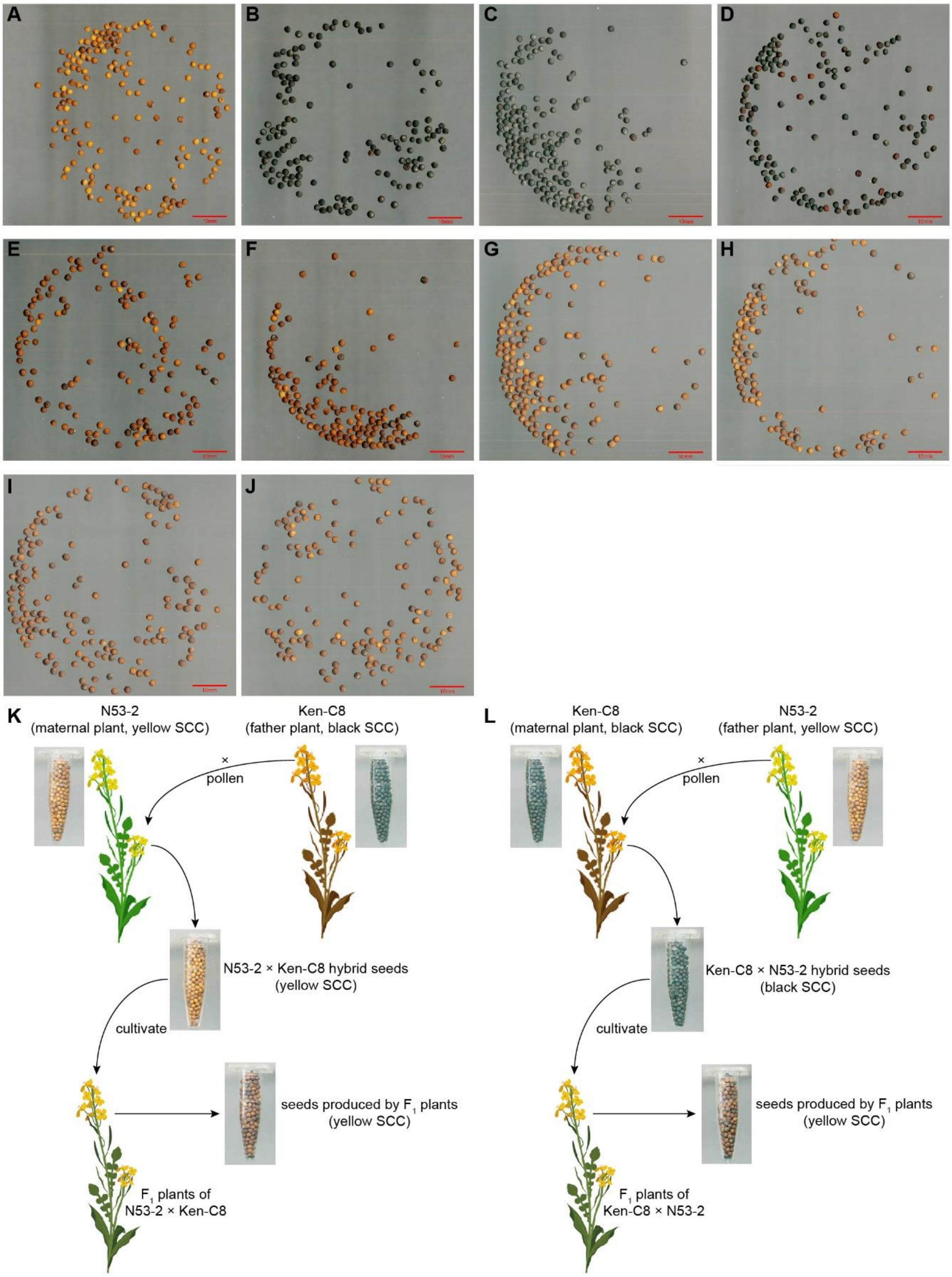

Fig. S1. The hybridization between N53-2 and black SCC *B. napus*.

The images of the matured seeds produced by N53-2 **(A)**, ZS11 **(B)**, J2016 **(C)**, Ken-C8 **(D)**, F_1 plants of ZS11 × N53-2 **(E)**, F_1 plants of N53-2 × ZS11 **(F)**, F_1 plants of J2016 × N53-2 **(G)**, F_1 plants of N53-2 × J2016 **(H)**, F_1 plants of Ken-C8 × N53-2 **(I)**, and F_1 plants of

N53-2 × Ken-C8 **(J)**. The red bars represent 10 mm. **(K, L)** The schematic diagram of the hybridization between N53-2 and black SCC *B. napus* lines (taken Ken-C8 for instance). The seed color of hybrid seeds of N53-2 × Ken-C8 **(K)** and Ken-C8 × N53-2 **(L)** align with that of the maternal plants, and both F_1 plants can produce yellow SCC seeds (F_2 seeds).

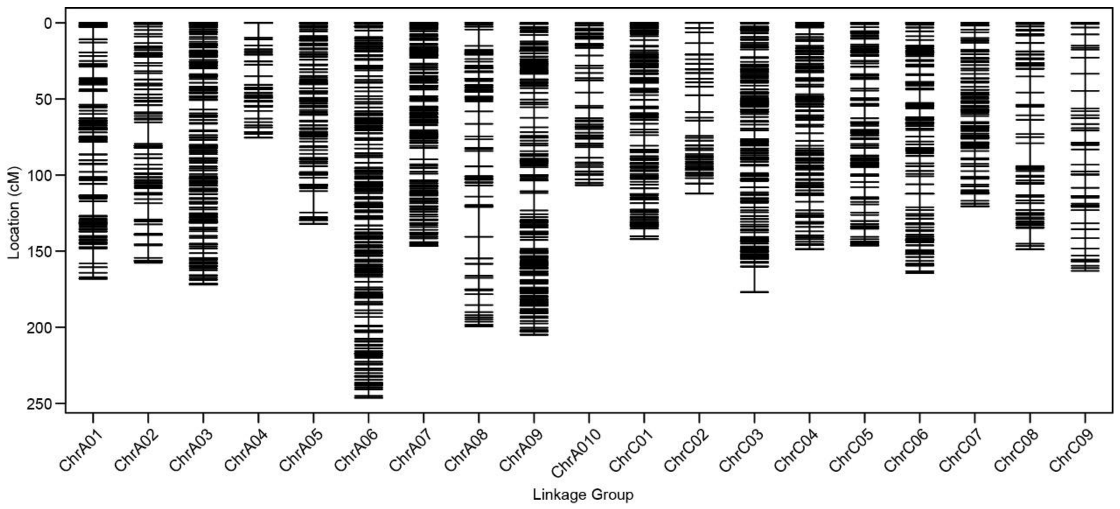

Fig. S2. The schematic diagram of the genetic linkage map.

The 19 linkage groups corresponded to 19 *B. napus* chromosomes, respectively. The parallel lines on each linkage group represent individual SNP markers, with their genetic position annotated on the left y-axis.

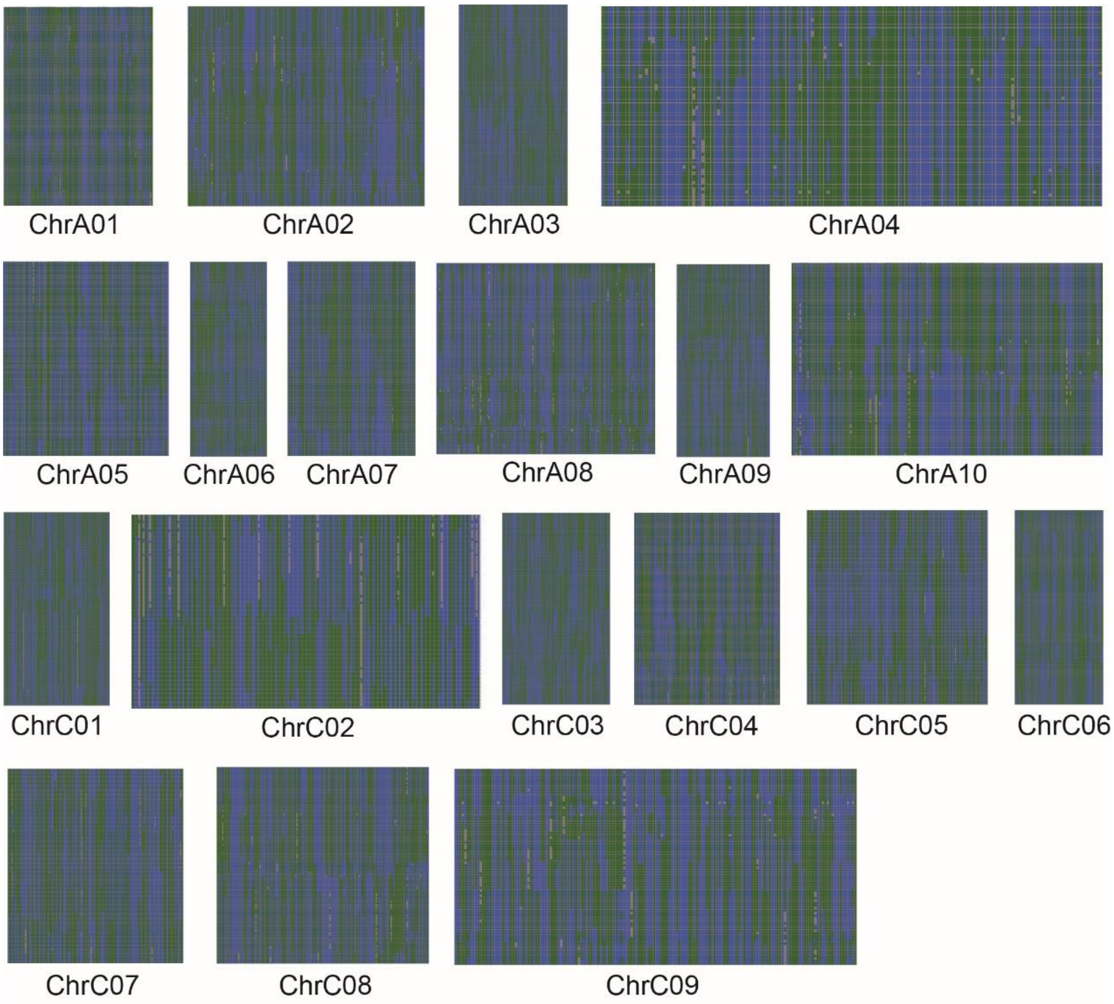

Fig. S3. The haplotype map of all 156 DH lines used for eQTL mapping in the present study.

Each row represents one marker, and each column corresponds to one DH line. Green squares indicate alleles from Ken-C8, blue squares indicate alleles from N53-2, white squares indicate undetermined alleles, and gray squares indicate missing data.

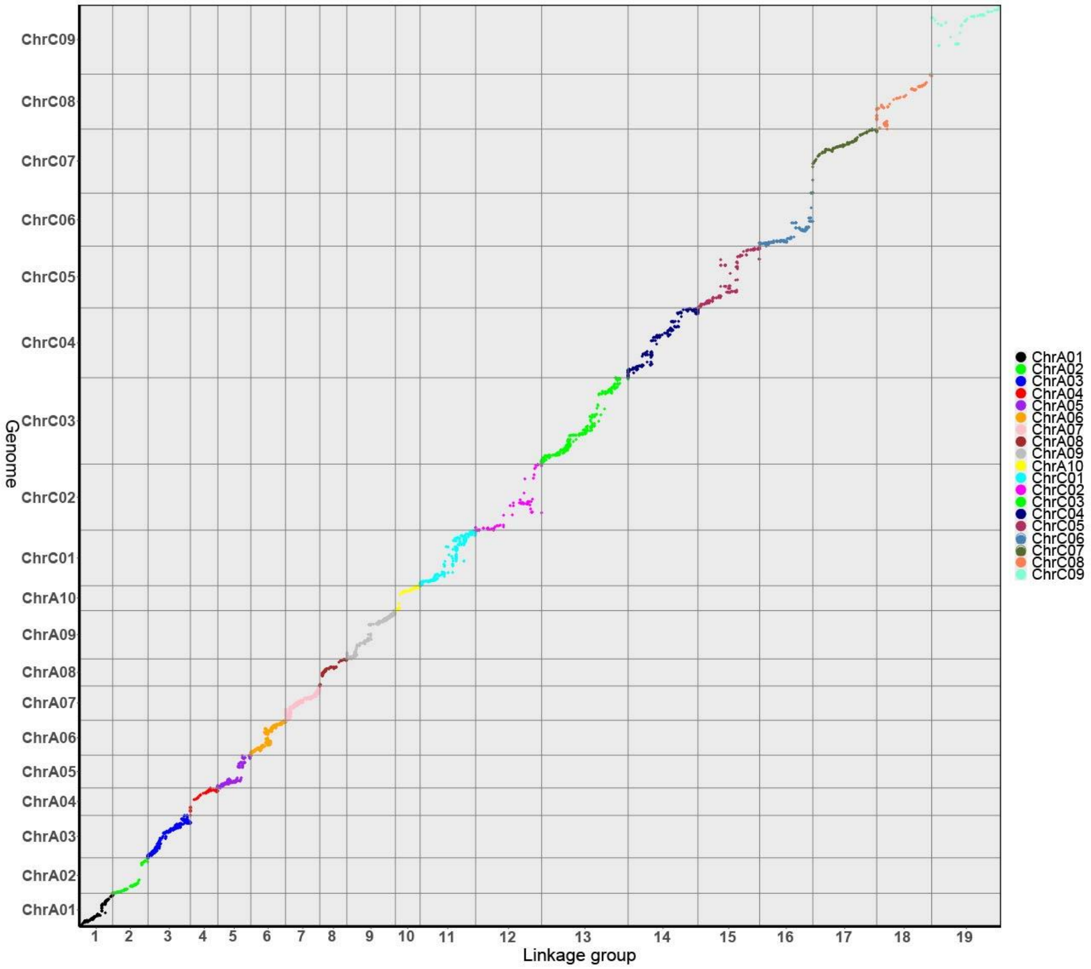

Fig. S4. The collinearity between the physical map and the genetic map.

The collinearity between the *B. napus* Darmor_*bzh* reference genome (left y-axis) and the genetic linkage map (x-axis). Different colors represent the markers in different linkage groups.

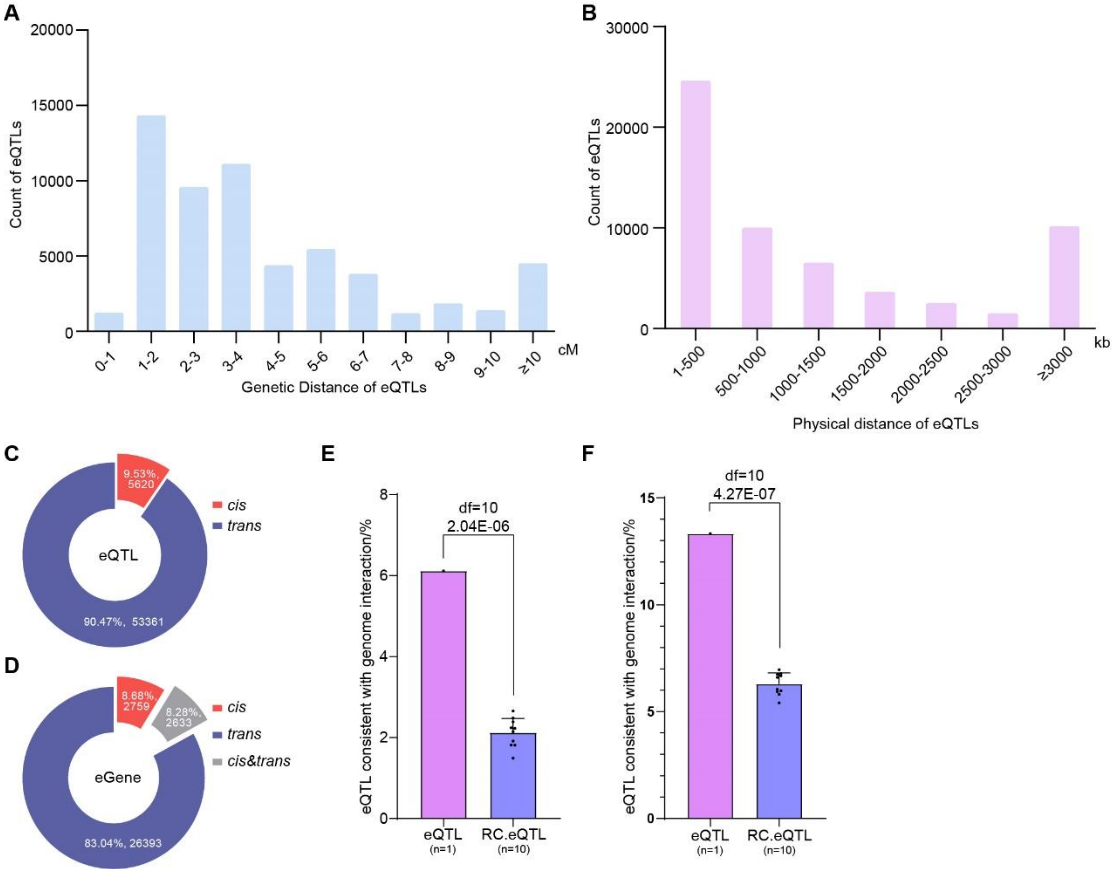

Fig. S5. The detailed characteristics of eQTLs.

The genetic **(A)** and physical **(B)** length distribution of eQTLs detected in the present study. **(C)** The number of *tran*-eQTL and *cis*-eQTL identified in the present study. **(D)** The number of eGenes regulated by *trans*-eQTL, *cis*-eQTL, or both. **(E, F)** The proportion of eQTLs and RC.eQTLs that aligned with the common **(E)** and unique **(F)** genome interactions of the two parents. The histograms represent the average values, and the error bars represent SD. The df and *p*-values are annotated above histograms (two-tailed one-way ANOVA). In addition, the number of replications is annotated behind the x-axis.

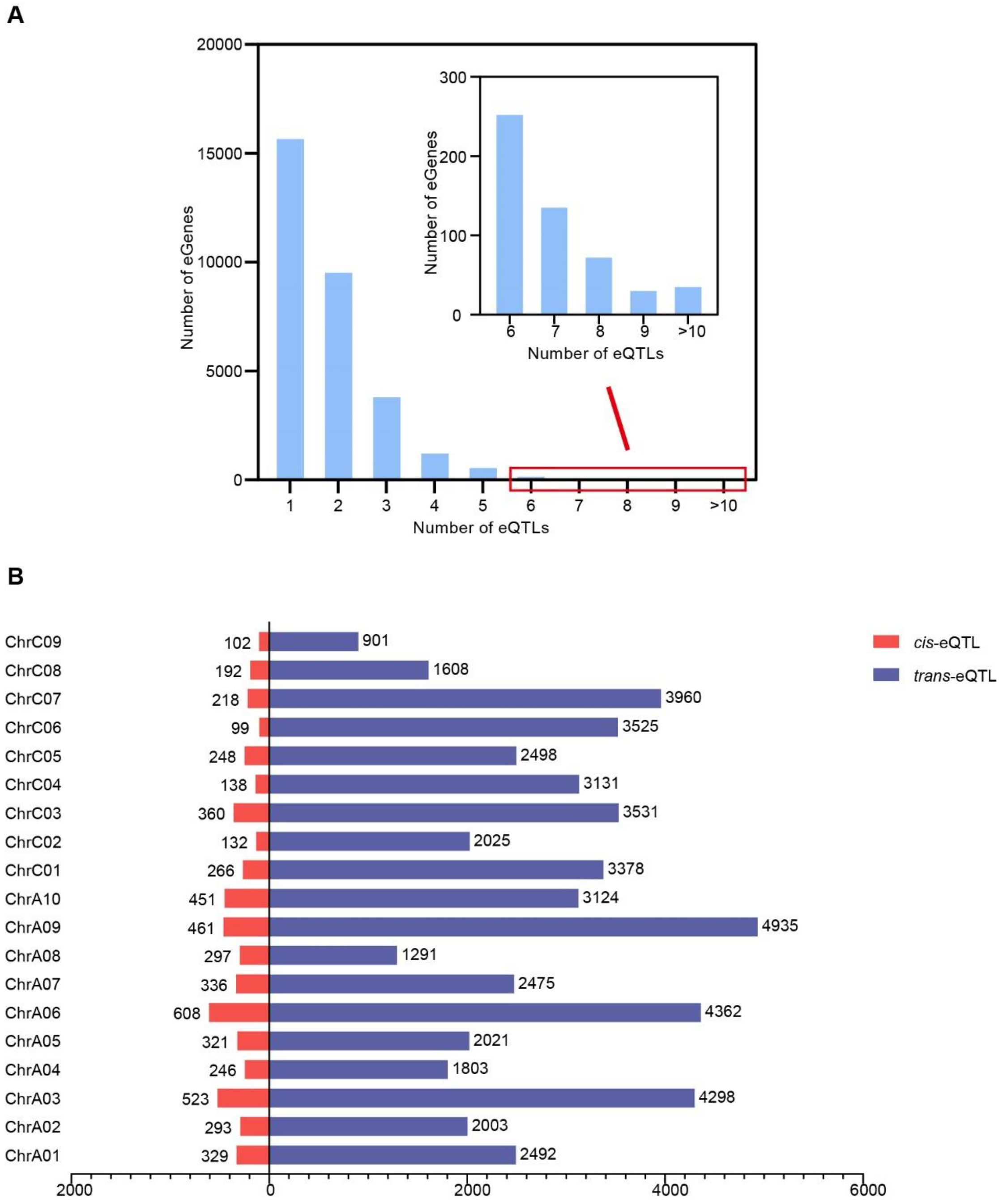

Fig. S6. The distributions of eQTLs.

(A) The distribution histogram of eQTL numbers per eGene. **(B)** The distribution histogram of *cis*-eQTLs and *trans*-eQTLs across 19 chromosomes.

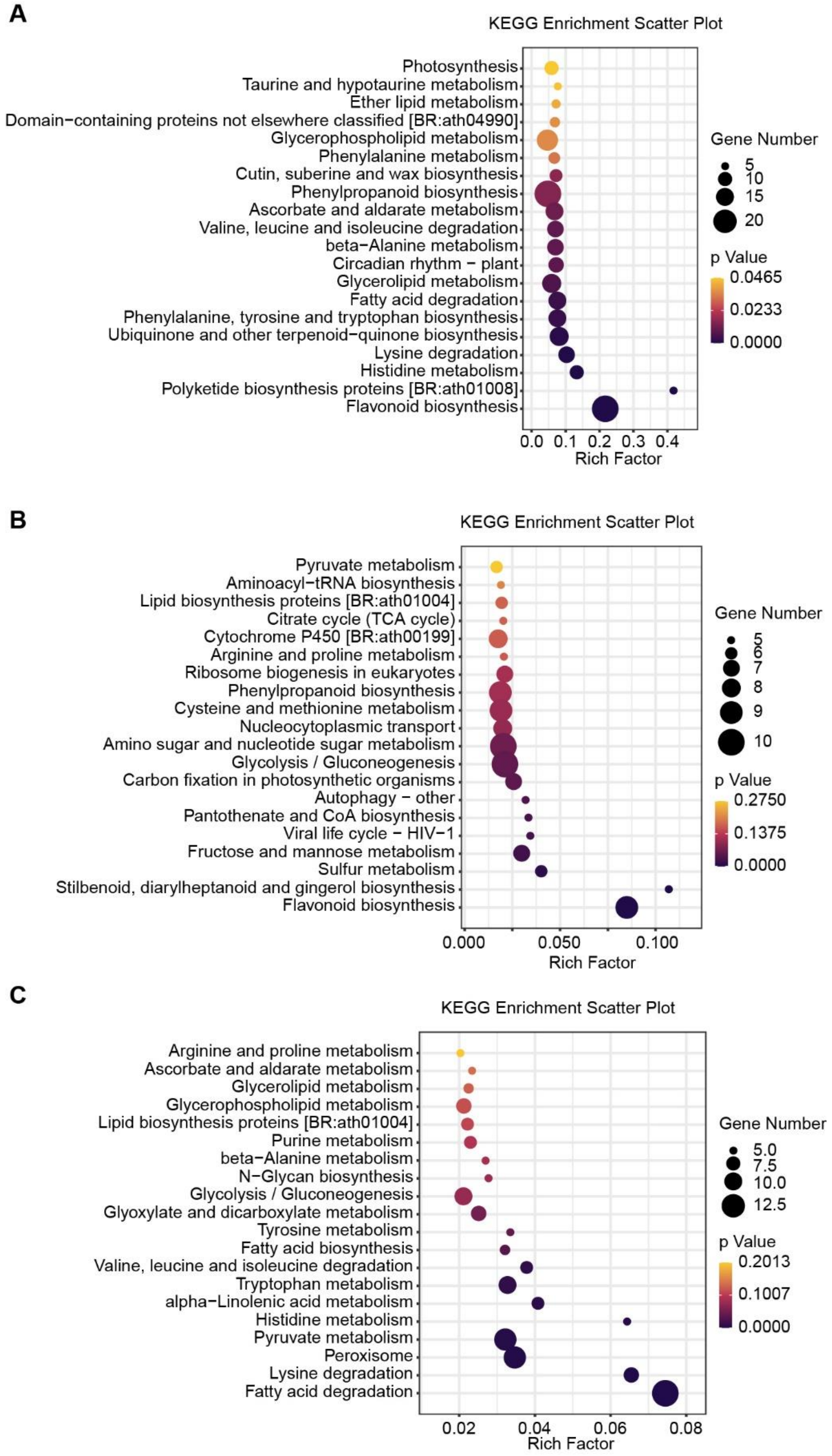

Fig. S7. The KEGG enrichment analysis of eGenes.

The KEGG enrichment analysis of *trans*-eQTL eGenes of Hotspot 13 **(A)**, Hotspot 6 **(B)**, and Hotspot 25 **(C)**.

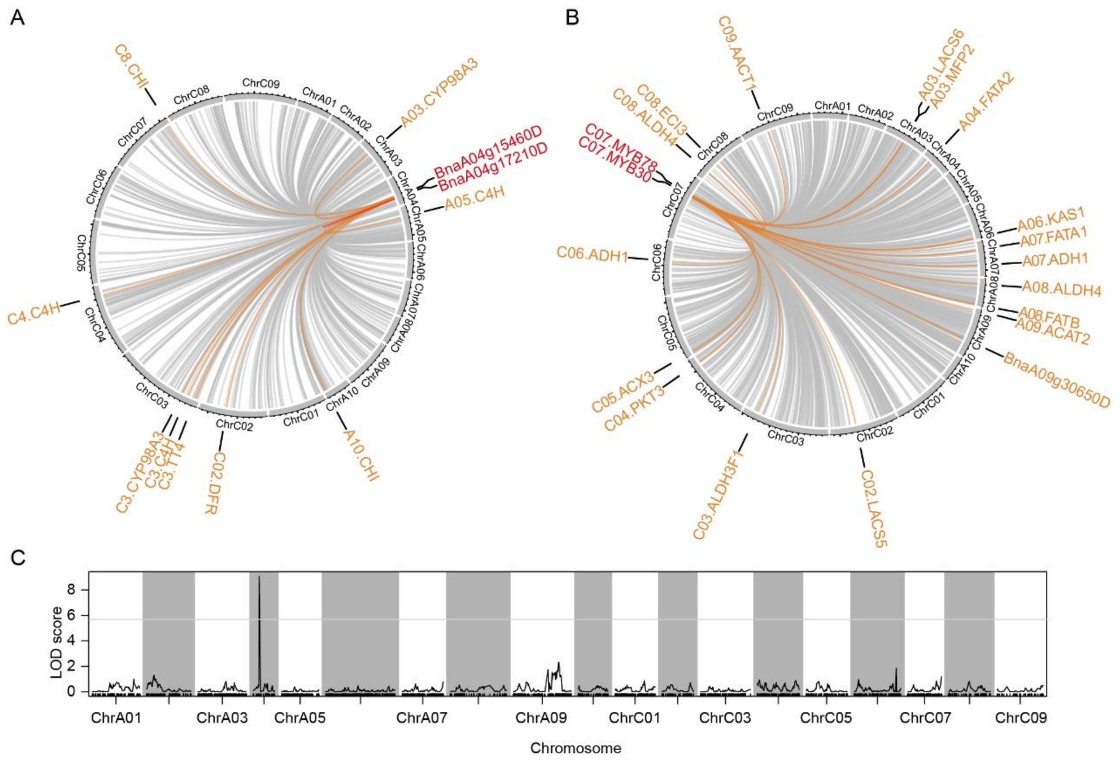

Fig. S8. The eQTL dristribution of Hotspot 6 and Hotspot 25.

(A) The *trans*-eQTLs located in Hotspot 6 and the position of their corresponding eGenes. The orange lines and labels represent the eGenes enriched in the flavonoid biosynthesis, and the red lines and labels represent the potential upstream regulator genes. **(B)** The *trans*-eQTLs located in Hotspot 25 and the position of their corresponding eGenes. The orange lines and labels represent the eGenes enriched in the fatty acid metabolic, and the red labels represent the potential upstream regulator genes. **(C)** The eQTL mapping of *BnaA04g17210D*.

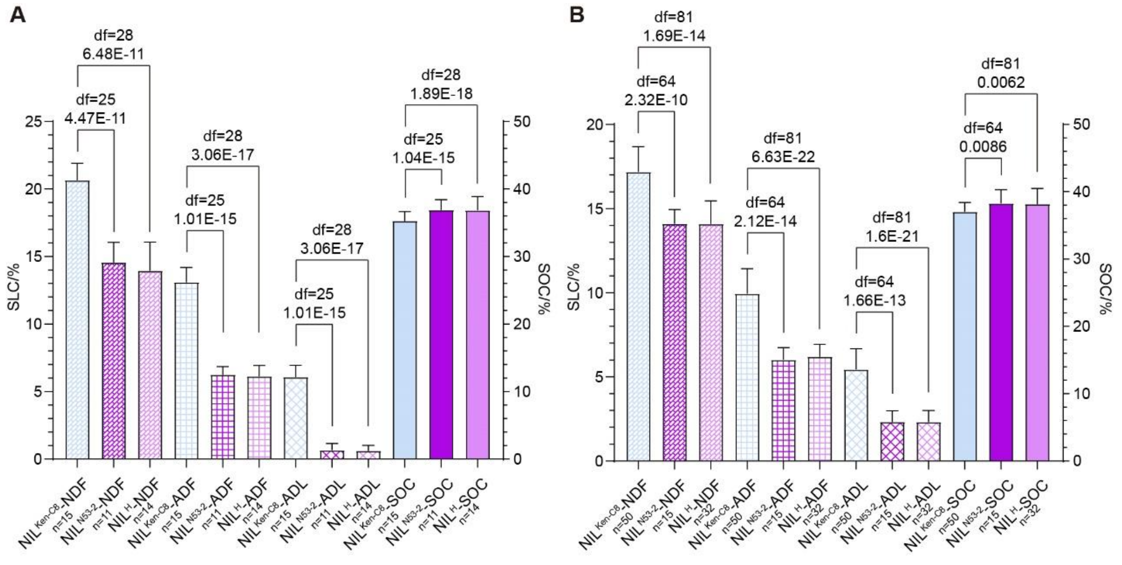

Fig. S9. The phenotype of different NIL groups.

The SOC and SLC of $NIL^{Ken\text{-}C8}$, $NIL^{N53\text{-}2}$, and NIL^{H} in Yangling (winter rape-producing area) **(A)** and Xining (spring rape-producing area) **(B)** in 2022. The histograms represent the average values, and the error bars represent the standard deviations (SD). The df and *p*-values were are annotated above histograms (two-tailed one-way ANOVA). In addition, the number of individuals in each group was is annotated behind the x-axis.

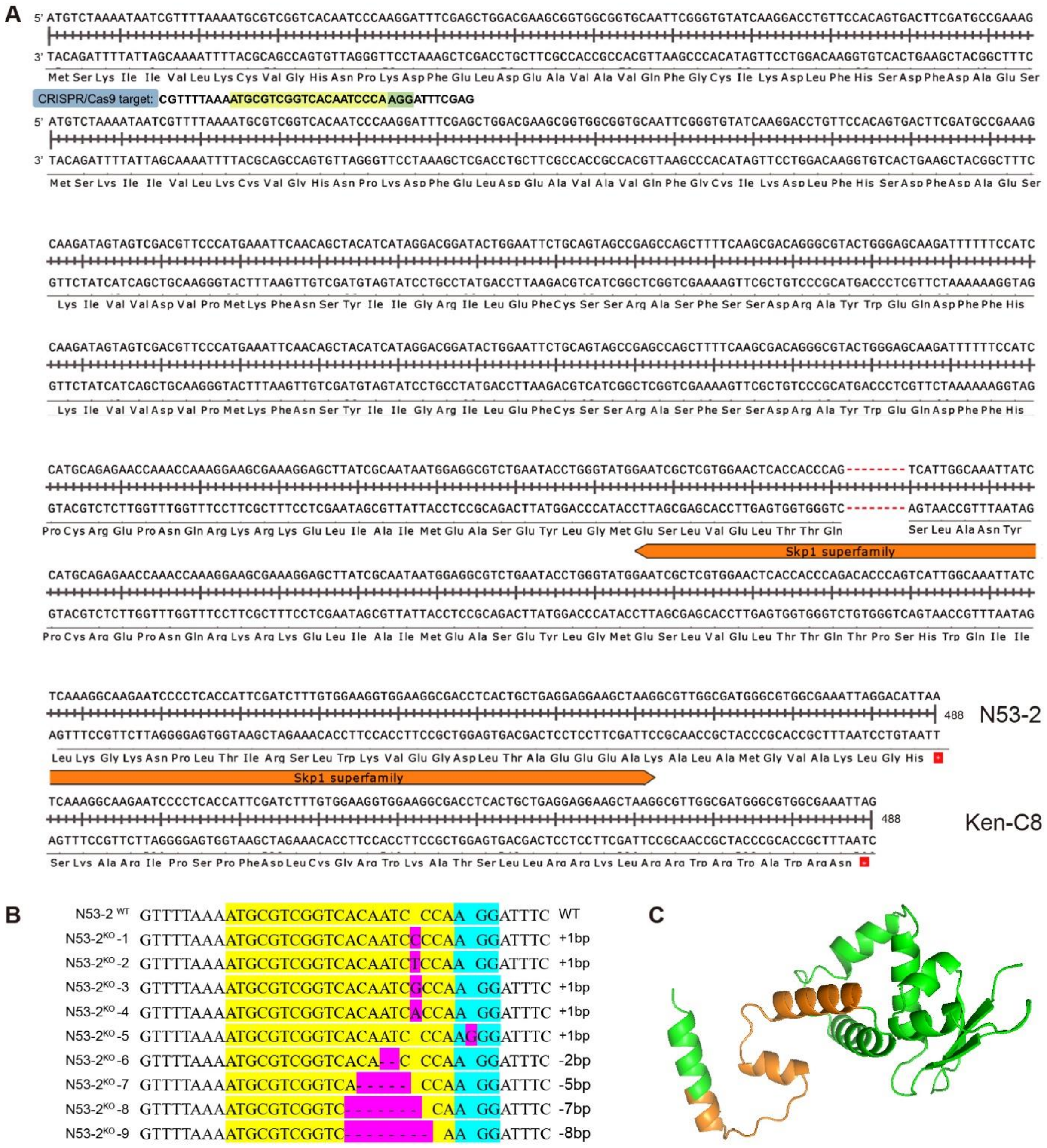

Fig. S10. The sequence and structure of DYSOC1.

(A) The sequence variation of *DYSOC1* between Ken-C8 and N53-2. The Skp1 domain and CRISPR/Cas9 target were labeled. **(B)** The mutation of *DYSOC1* in the nine N53-2^{KO} lines. **(C)** The predicted tertiary structure of DYSOC1 in N53-2, and the predicted Skp1 domain was annotated in orange.

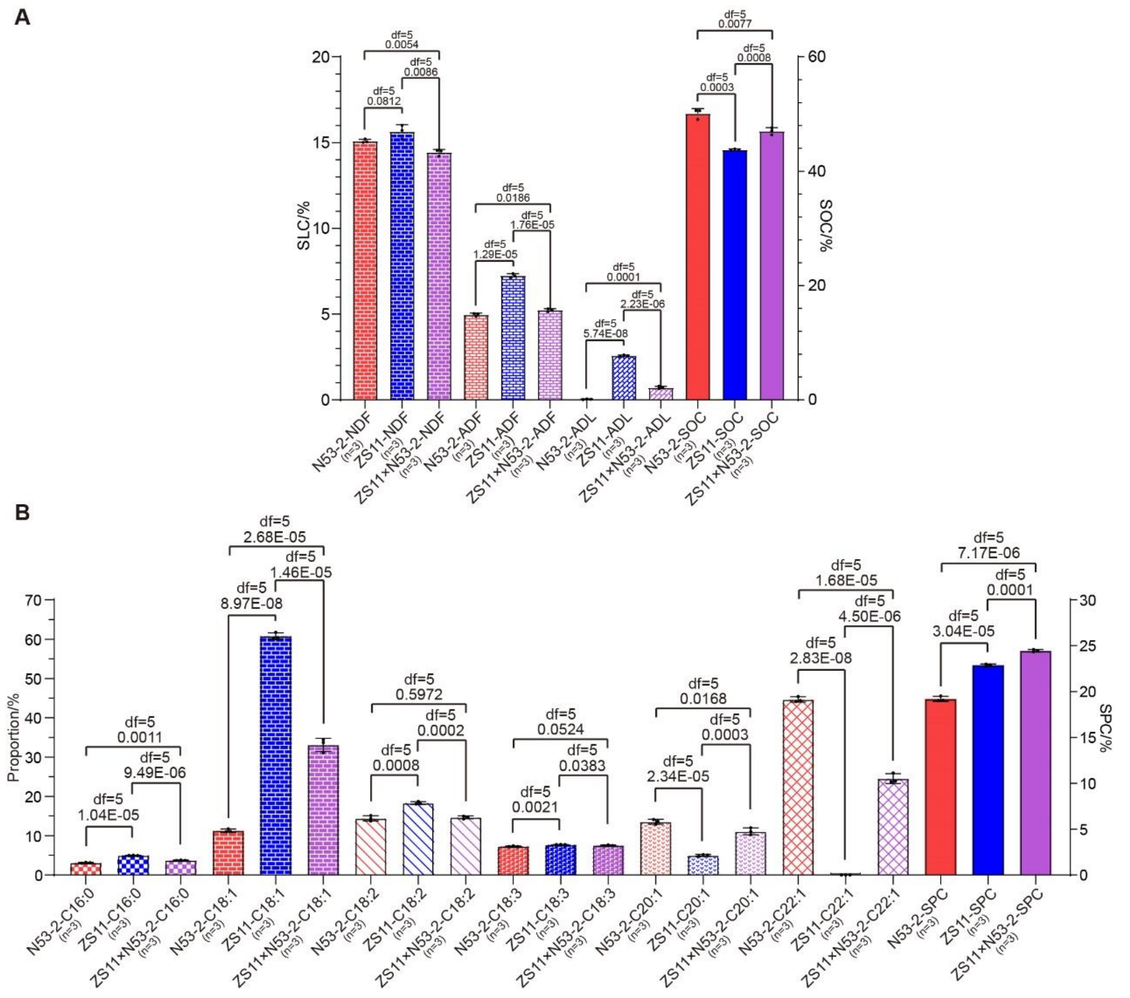

Fig. S11. The phenotype variation of ZS11 and N53-2.

(A) The SLC (left y-axis) and SOC (right y-axis) of seeds produced by ZS11, N53-2, and the F_1 plants of ZS11 × N53-2. Each point in the histogram represents one repetition, the histograms represent the average values, and the error bars represent SD. The df and *p*-values are annotated above histograms (two-tailed one-way ANOVA). In addition, the number of replications is annotated behind the x-axis. **(B)** The FA proportions (left y-axis) and SPC (left y-axis) of seeds produced by ZS11, N53-2, and the F_1 plants of ZS11 × N53-2. Each point in the histogram represents one repetition, the histograms represent the average values, and the error bars represent the SD. The df and *p*-values are annotated above histograms (two-tailed one-way ANOVA). In addition, the number of replications is annotated behind the x-axis.

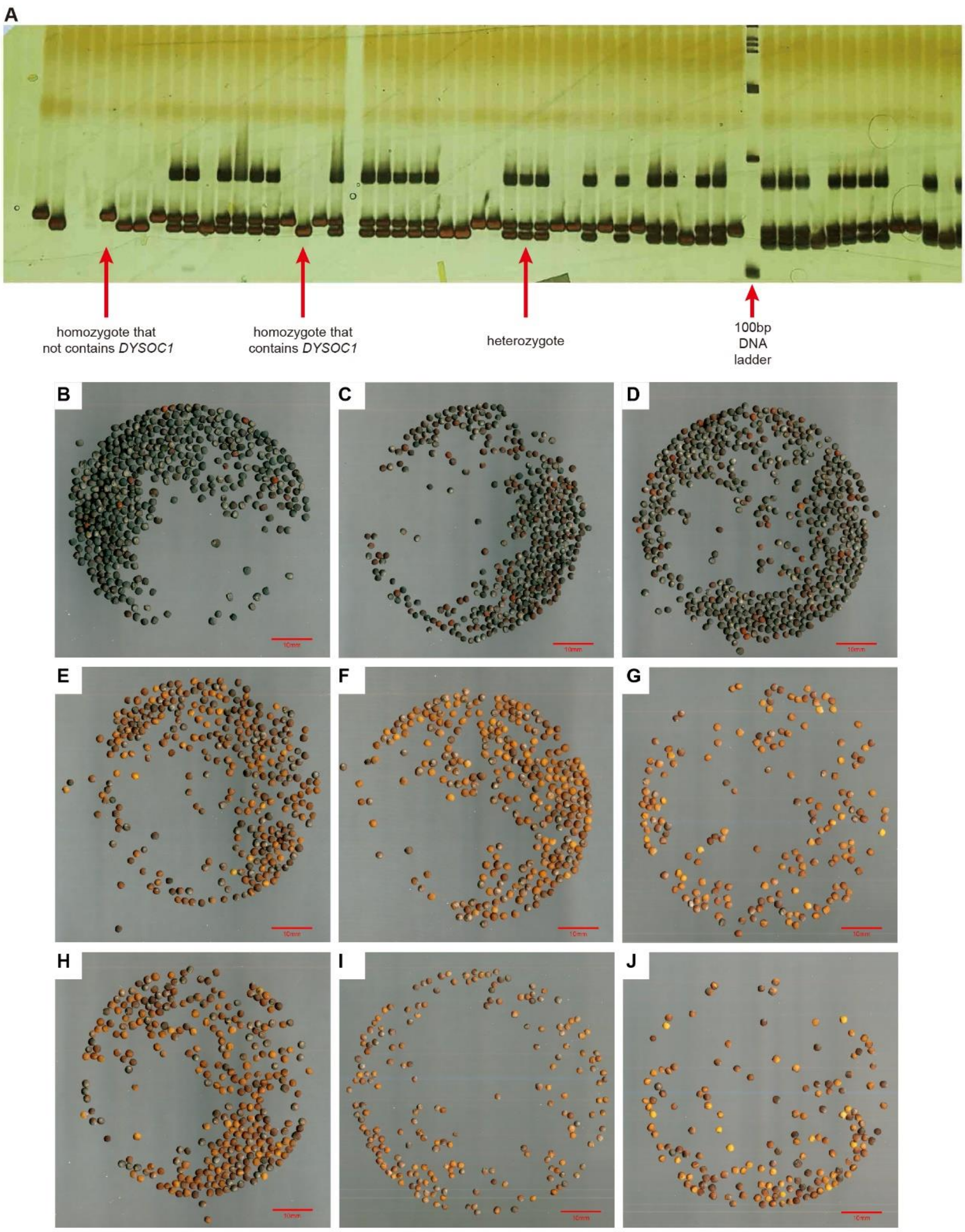

Fig. S12. The seed color variation of different F_2 groups.

(A) Genotyping of the F_2 population individuals derived from the hybridization of ZS11 and N53-2 by Ms-4. The mature seeds of F_2^{ZS11} **(B-D)**, $F_2^{N53\text{-}2}$ **(E-G)**, and F_2^{H} **(H-J)**. The red bars represent 10 mm.

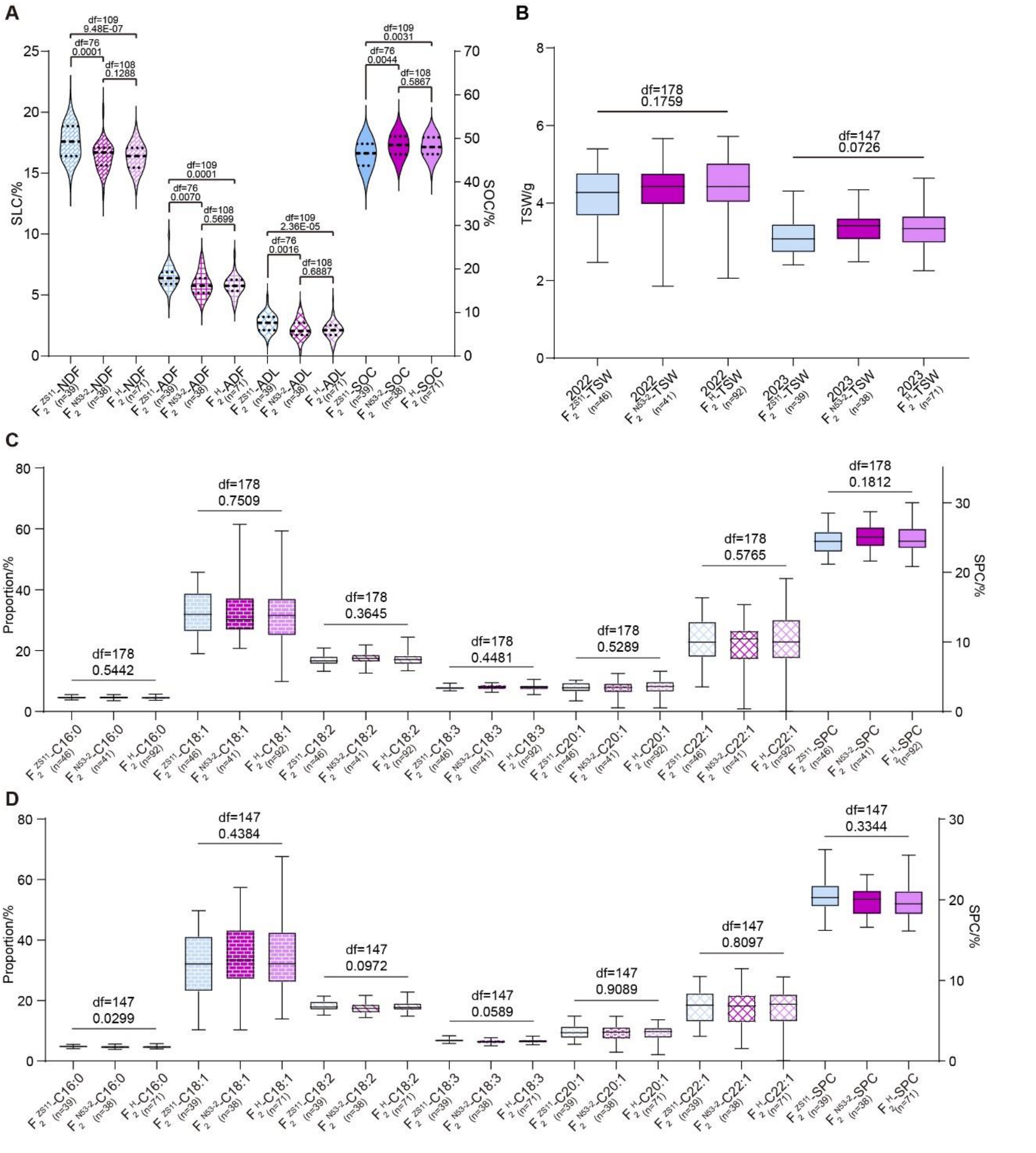

Fig. S13. The phenotype variation of different F_2 groups.

(A) The SLC (left y-axis) and SOC (right y-axis) of F_2^{ZS11}, F_2^{N53-2}, and F_2^{H} in the 23WH microenvironment. The upper, middle, and lower horizontal dashed lines of violin plots represent the first, second, and third quartiles of each group, respectively. The df and *p*-values are annotated above histograms (two-tailed one-way ANOVA). In addition, the number of individuals of F_2^{ZS11}, F_2^{N53-2}, and F_2^{H} are annotated behind the x-axis. **(B)** The TSW of F_2^{ZS11}, F_2^{N53-2}, and F_2^{H} in the 22WH (left three) and 23WH (right three) microenvironments. The error bars represent the Min and Max values of each group, and the upper, middle, and lower parallel lines represent the first, second, and third quartiles of each group, respectively. The df and *p*-values are annotated above histograms (two-tailed one-way ANOVA). In addition, the individual numbers of each group are annotated behind the x-axis. **(C-D)** The box plots of the major FA proportions (left y-axis) and SPC (right y-axis) of F_2^{ZS11}, F_2^{N53-2}, and F_2^{H} in the 22WH

(C) and 23WH **(D)** microenvironments. The error bars represent the Min and Max values of each group, and the upper, middle, and lower parallel lines represent the first, second, and third quartiles of each group, respectively. The df and p-values are annotated above histograms (two-tailed one-way ANOVA). In addition, the individual numbers of each group are annotated behind the x-axis.

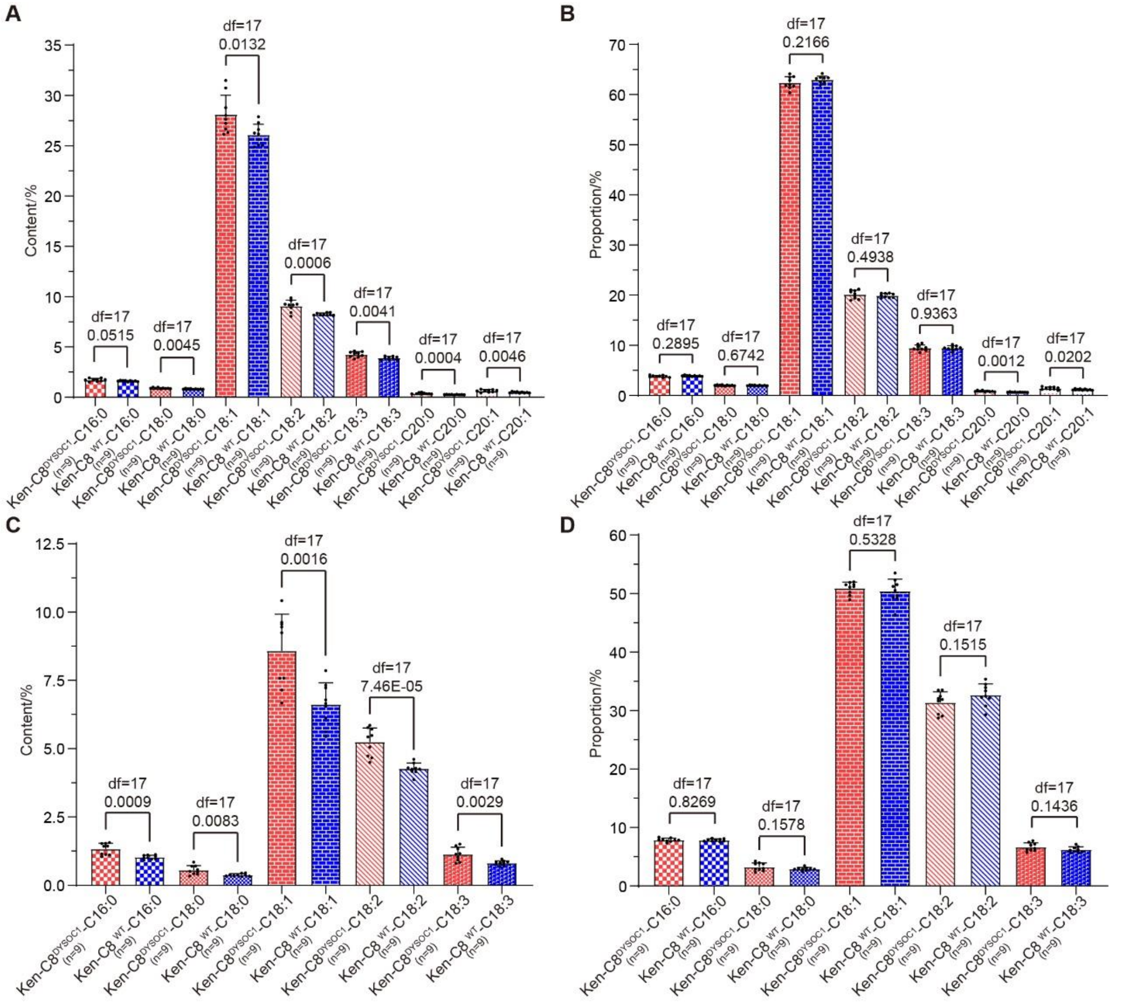

Fig. S14. The fatty acid content and proportion of Ken-C8 transgenic lines.

The content and proportion of FA composition in the embryo **(A, B)** and seed coat **(C, D)** of Ken-C8 transgenic lines. Each point in the histogram represents one independent transgenic line, the histograms represent the average values, and the error bars represent SD. The df and *p*-values are annotated above histograms (two-tailed one-way ANOVA). In addition, the number of independent lines is annotated behind the x-axis. Three repetitions were conducted for each line, and the average trait value was adopted.

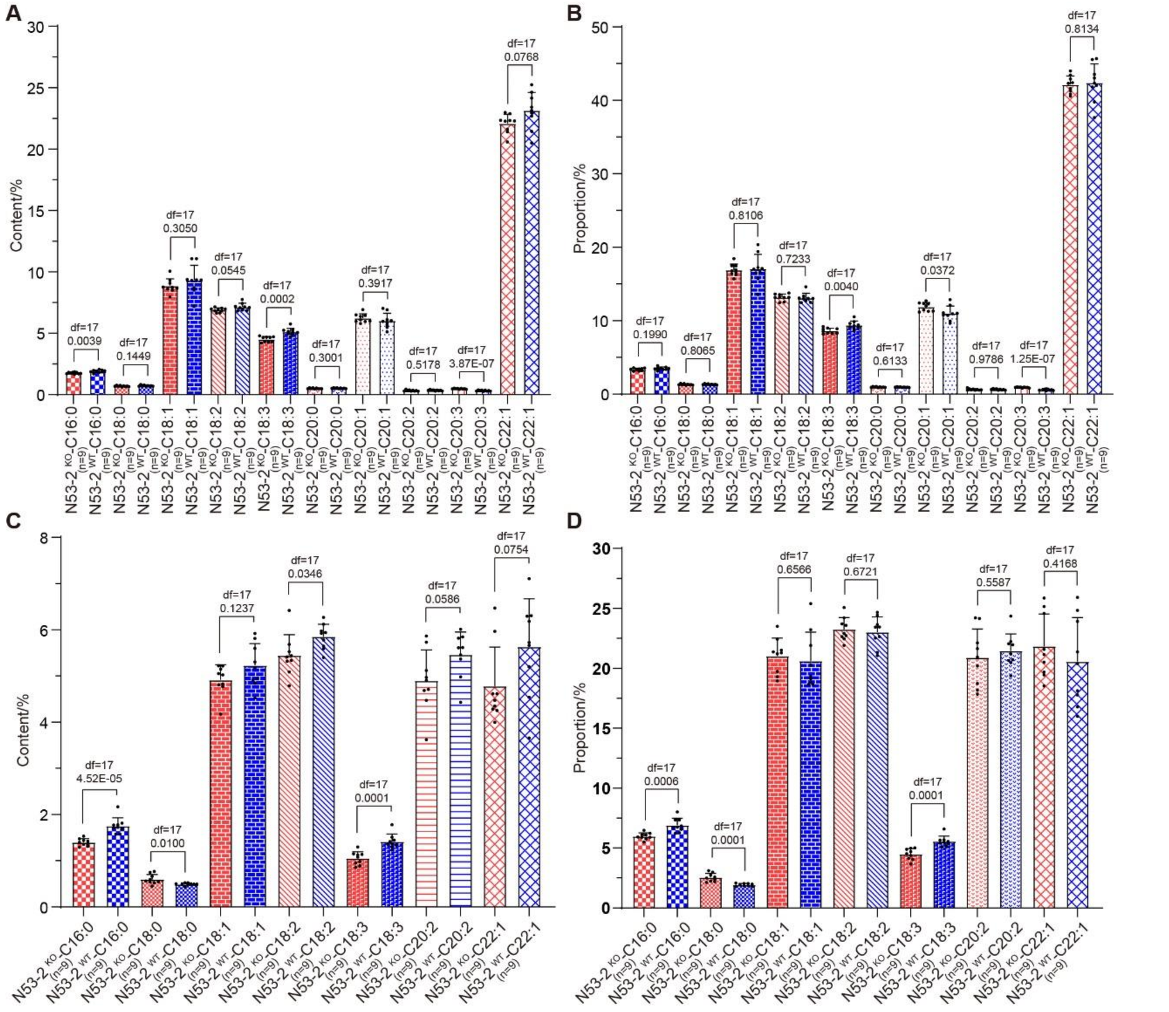

Fig. S15. The fatty acid content and proportion of N53-2 transgenic lines.

The content and proportion of FA composition in the embryo **(A, B)** and seed coat **(C, D)** of N53-2 transgenic lines. Each point in the histogram represents one independent transgenic line, the histograms represent the average values, and the error bars represent SD. The df and *p*-values are annotated above histograms (two-tailed one-way ANOVA). In addition, the number of independent lines is annotated behind the x-axis. Three repetitions were conducted for each line, and the average trait value was adopted.

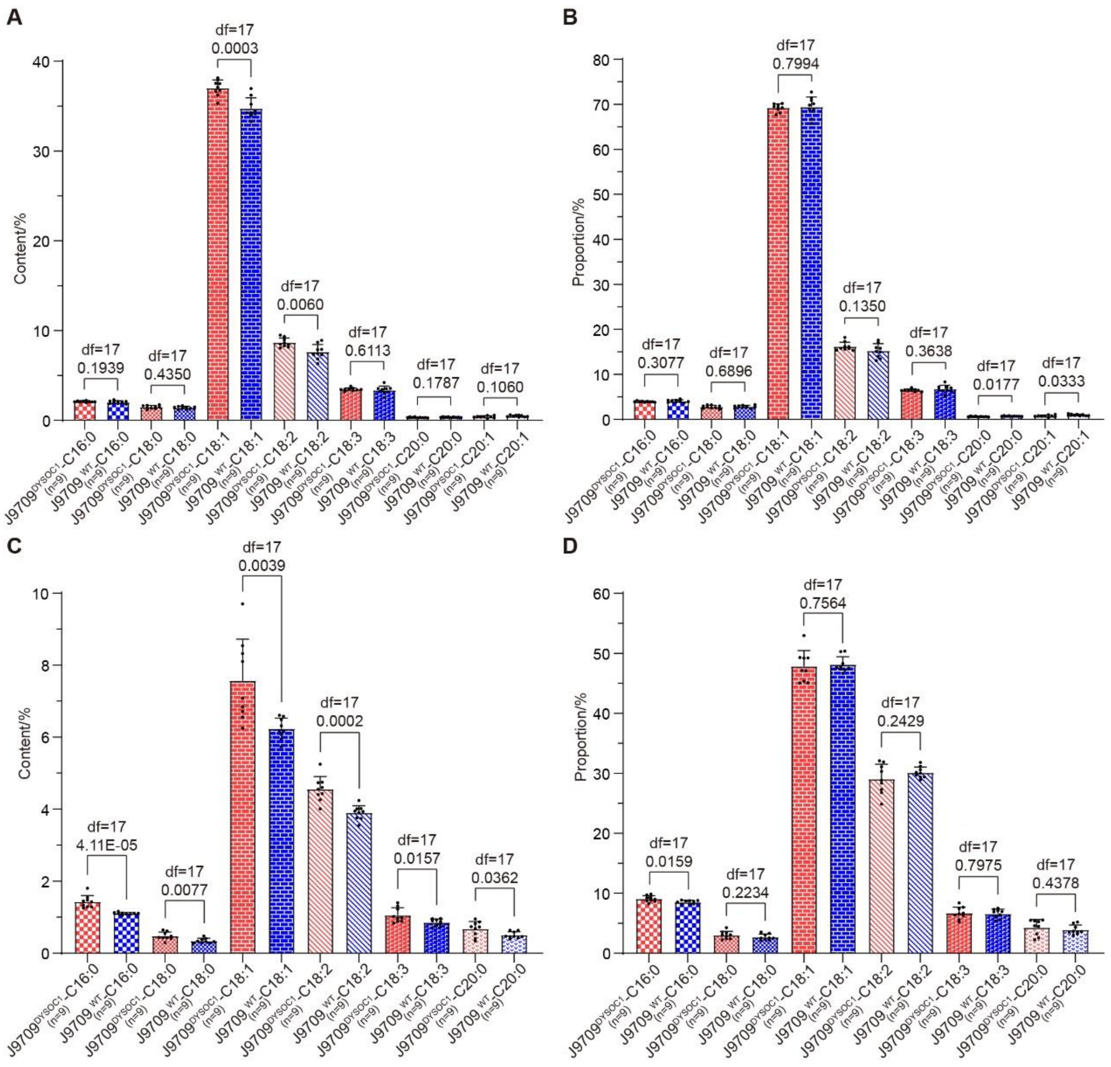

Fig. S16. The fatty acid content and proportion of J9709 transgenic lines.

The content and proportion of FA composition in the embryo **(A, B)** and seed coat **(C, D)** of J9709 transgenic lines. Each point in the histogram represents one independent transgenic line, the histograms represent the average values, and the error bars represent SD. The df and *p*-values are annotated above histograms (two-tailed one-way ANOVA). In addition, the number of independent lines is annotated behind the x-axis. Three repetitions were conducted for each line, and the average trait value was adopted.

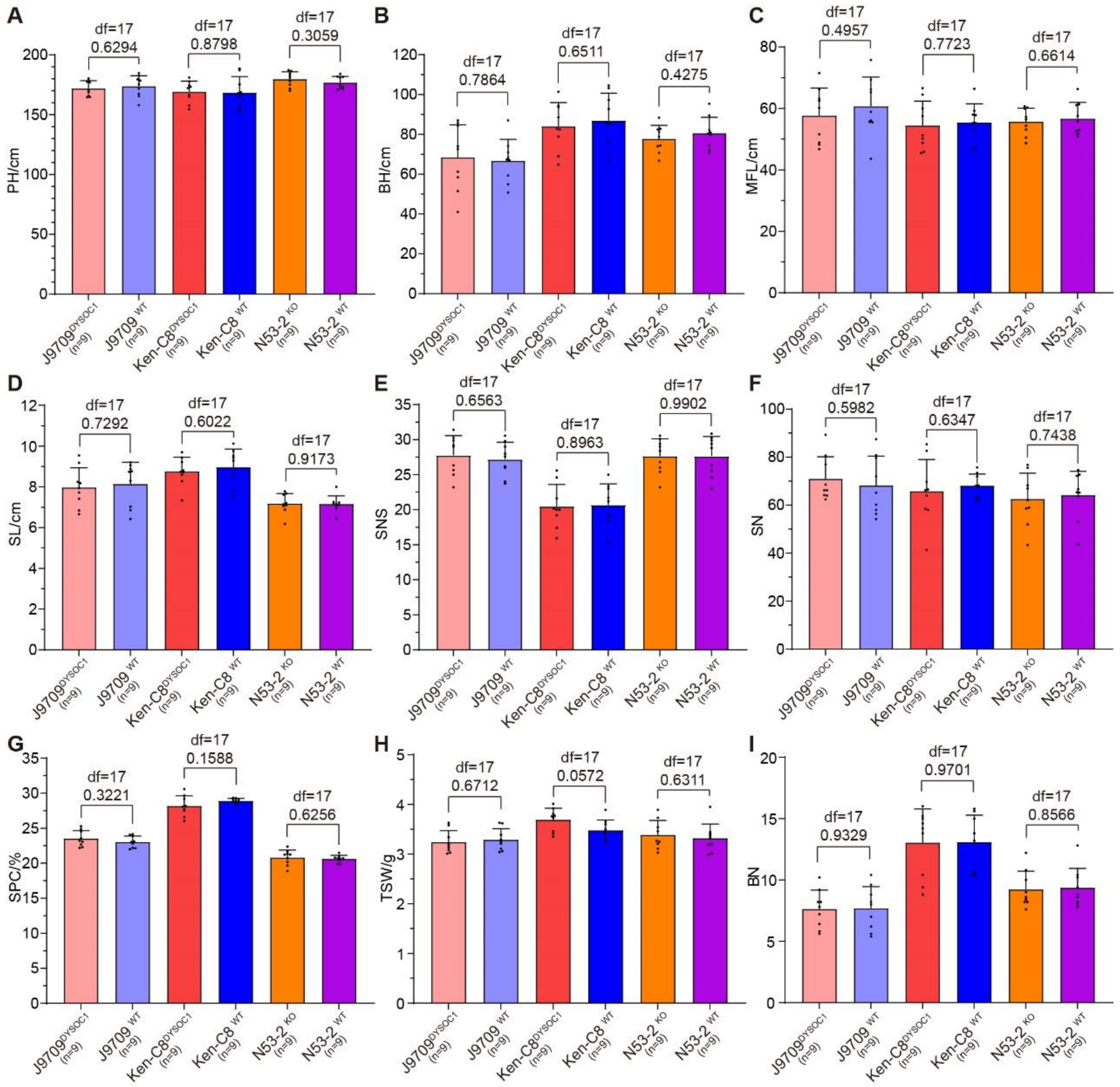

Fig. S17. The plant architecture of transgenic lines.

The PH **(A)**, BH **(B)**, MFL **(C)**, SL **(D)**, SNS **(E)**, SN **(F)**, SPC **(G)**, TSW **(H)**, and BN **(I)** of Ken-C8, N53-2, and J9709 transgenic lines. Each point in the histogram represents one independent transgenic line, the histograms represent the average values, and the error bars represent SD. The df and *p*-values are annotated above histograms (two-tailed one-way ANOVA). In addition, the number of independent lines is annotated behind the x-axis. For plant architecture traits, five healthy individuals from each line were randomly selected for phenotype measurement, and the average value was adopted. For seed traits, five healthy individuals from each line were randomly selected, and their matured seeds were pooled for trait measurement, with three repetitions. The average value was adopted.

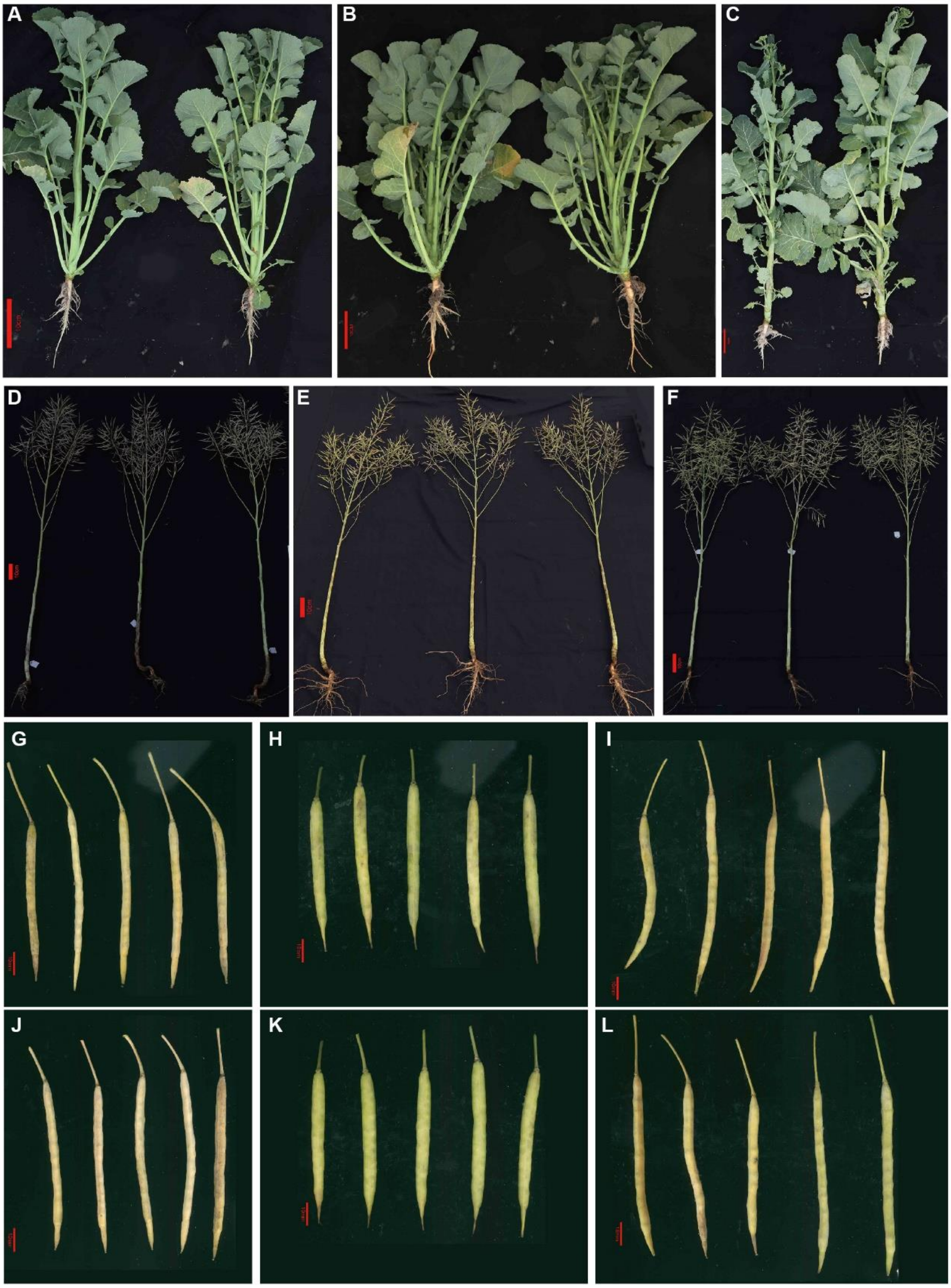

Fig. S18. The morphology of transgenic lines.

(A) The seedlings of Ken-C8^{WT} (left) and Ken-C8^{DYSOC1} (right). **(B)** The seedlings of N53-2^{WT} (left) and N53-2^{KO} (right). **(C)** The seedlings of J9709^{WT} (left) and J9709^{DYSOC1} (right). **(D)** The mature plants of Ken-C8^{WT} (left) and Ken-C8^{DYSOC1} (middle and right). **(E)** The mature plants of N53-2^{WT} (left) and N53-2^{DYSOC1} (middle and right). **(F)** The mature plants of J9709^{WT} (left) and J9709^{DYSOC1} (middle and right). The siliques of Ken-C8^{WT} **(G)**, N53-2^{WT} **(H)**, J9709^{WT} **(I)**, Ken-C8^{DYSOC1} **(J)**, N53-2^{KO} **(K)**, and J9709^{DYSOC1} **(L)**. The red bars in **(A-F)**

represent 10 cm, and the red bars in **(G-L)** represent 10 mm.

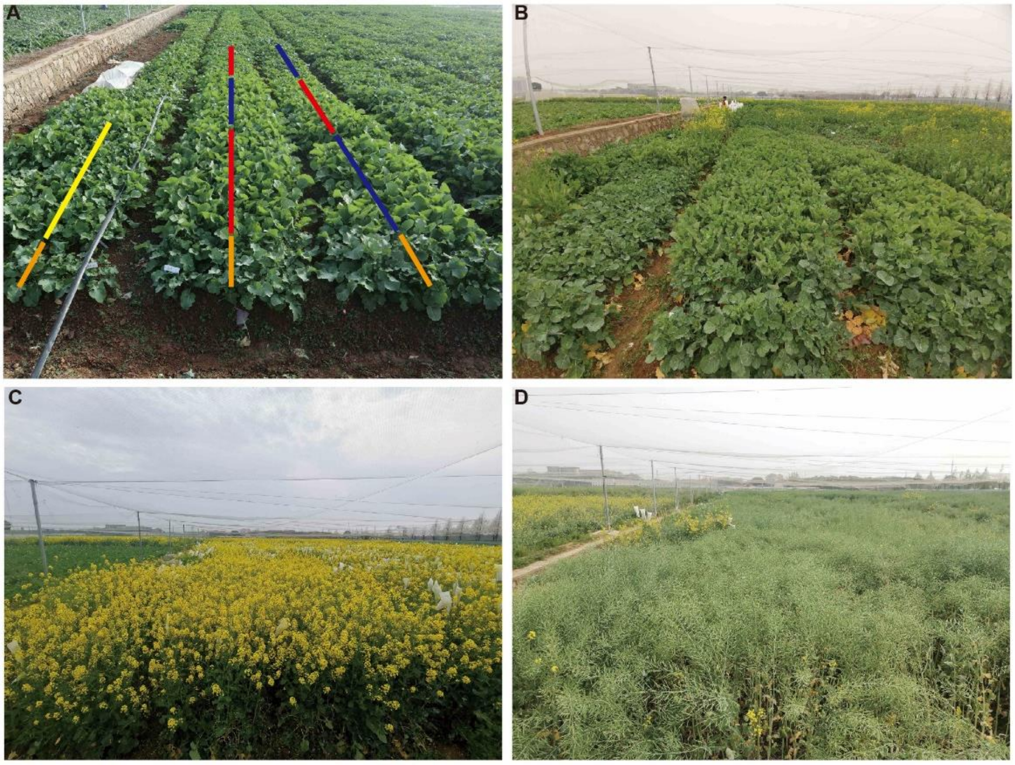

Fig. S19. The plot yield experiment of NIL$^{Ken\text{-}C8}$ and NIL$^{N53\text{-}2}$.

The images of the plot yield experiment of NIL$^{Ken\text{-}C8}$ and NIL$^{N53\text{-}2}$ at different stages: seedling stage **(A)**, bolting stage **(B)**, flowing stage **(C)**, and immature stage **(D)**. In **(A)**, the red lines represent NIL $^{N53\text{-}2}$ plots, the blue lines represent NIL $^{Ken\text{-}C8}$ plots, the yellow line represents N53-2 plot, and the orange lines represent the guard rows.

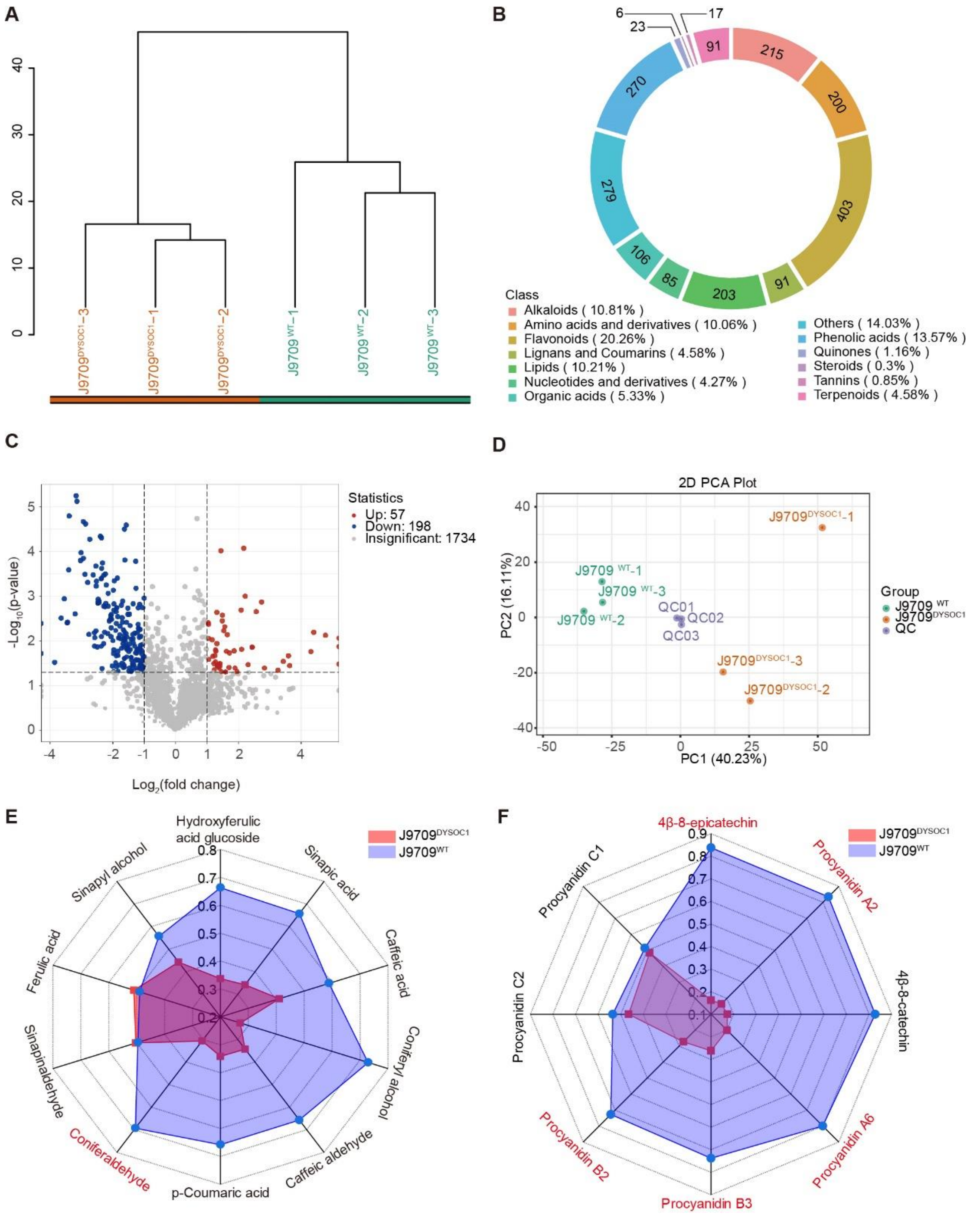

Fig. S20. The widely targeted metabolomic analysis of J9709^{DYSOC1} and J9709^{WT}.

The cluster tree **(A)**, metabolites classification **(B)**, DAM analysis **(C)**, and PCA **(D)** of the widely targeted metabolomic analysis (QC indicates quality control group). **(E)** The content of detected metabolites that take part in lignin monomers biosynthesis in the seed coat of J9709^{DYSOC1} and J9709^{WT}. **(F)** The content of detected PAs in the seed coat of J9709^{DYSOC1} and J9709^{WT}. The content of metabolites has been normalized in (E, F). For example, “normalized coniferaldehyde content in J9709^{DYSOC1}” = “average coniferaldehyde content in J9709^{DYSOC1}”

/ (“average coniferaldehyde content in J9709^{DYSOC1}” + “average coniferaldehyde content in J9709^{WT}”). The DAMs were highlighted in red color in **(E, F)**.

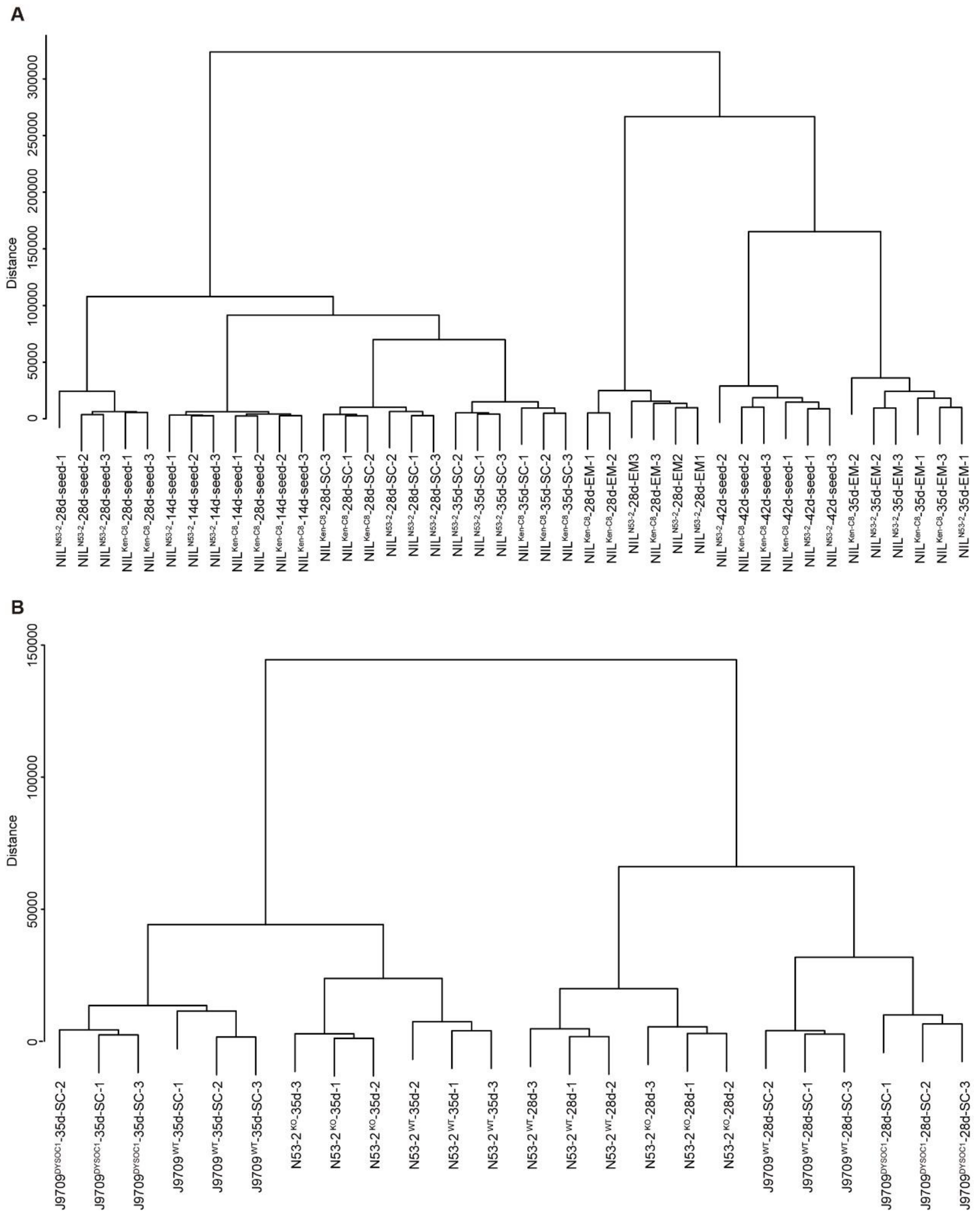

Fig. S21. The cluster tree of the RNA-seq samples.

The cluster tree of the RNA-seq samples of NIL **(A)** and transgenic materials **(B)**.

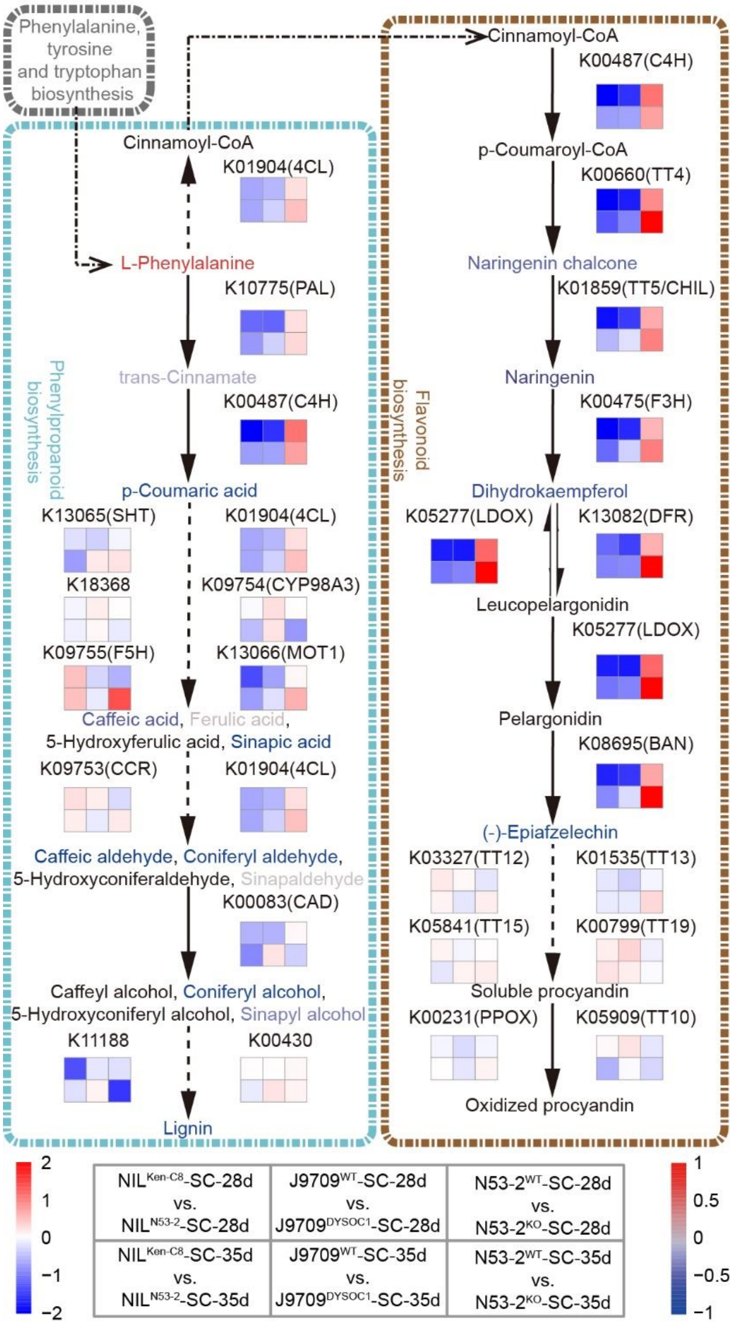

Fig. S22. The pathway of lignin monomers and PA synthesis in *B. napus*.

The heat maps (labeled at the bottom left) were drowned by $\log_2$(fold change) of the corresponding KEGG orthologies between different samples (annotated at the bottom center). The colors of metabolites (labeled at the bottom right) represent the $\log_2$(fold change) of their content in the seed coat of J9709^{WT} vs. J9709^{DYSOC1}.

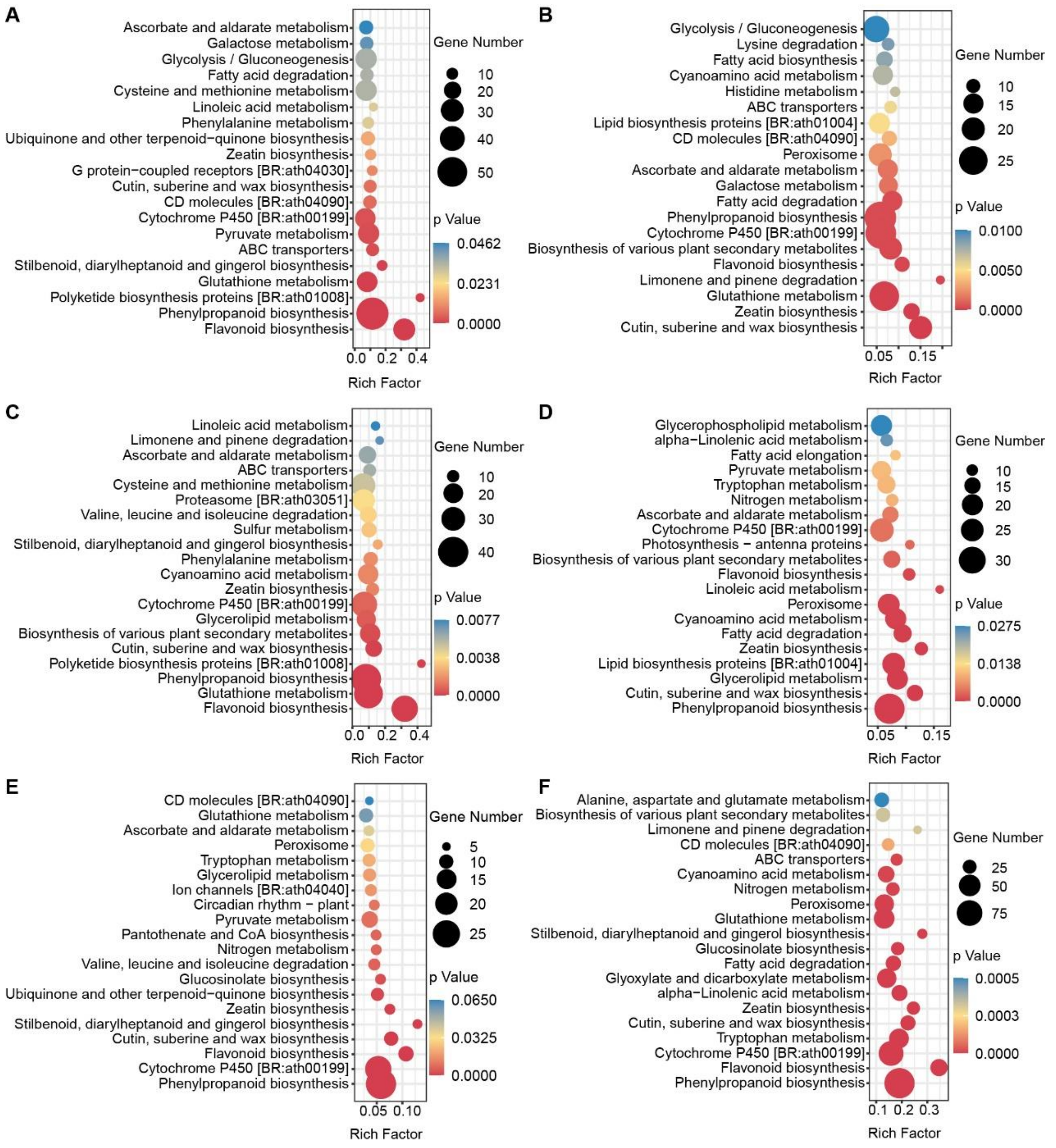

Fig. S23. The KEGG enrichment analysis of DEGs in NIL and transgenic materials.

The KEGG enrichment analysis of the DEGs of $NIL^{Ken\text{-}C8}$-28d-SC vs. $NIL^{N53\text{-}2}$-28d-SC **(A)**, $NIL^{Ken\text{-}C8}$-35d-SC vs. $NIL^{N53\text{-}2}$-35d-SC **(B)**, $J9709^{WT}$-28d-SC vs. $J9709^{DYSOC1}$-28d-SC **(C)**, $J9709^{WT}$-35d-SC vs. $J9709^{DYSOC1}$-35d-SC **(D)**, N53-2^{KO}-28d-SC vs. N53-2^{WT}-28d-SC **(E)**, and N53-2^{KO}-35d-SC vs. N53-2^{WT}-35d-SC **(F)**.

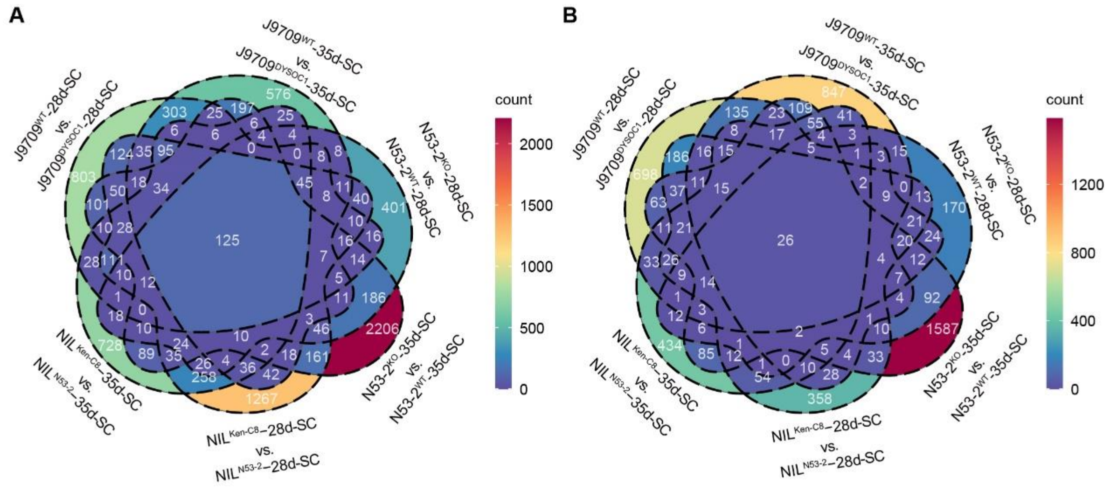

Fig. S24. The Venn diagram of DEGs in NIL and transgenic materials.

The Venn diagram of upregulated DEGs **(A)** and downregulated DEGs **(B)** of NIL$^{Ken\text{-}C8}$-28d-SC vs. NIL$^{N53\text{-}2}$-28d-SC, NIL$^{Ken\text{-}C8}$-35d-SC vs. NIL$^{N53\text{-}2}$-35d-SC, J9709^{WT}-28d-SC vs. J9709^{DYSOC1}-28d-SC, J9709^{WT}-35d-SC vs. J9709^{DYSOC1}-35d-SC, N53-2^{KO}-28d-SC vs. N53-2^{WT}-28d-SC, and N53-2^{KO}-35d-SC vs. N53-2^{WT}-35d-SC.

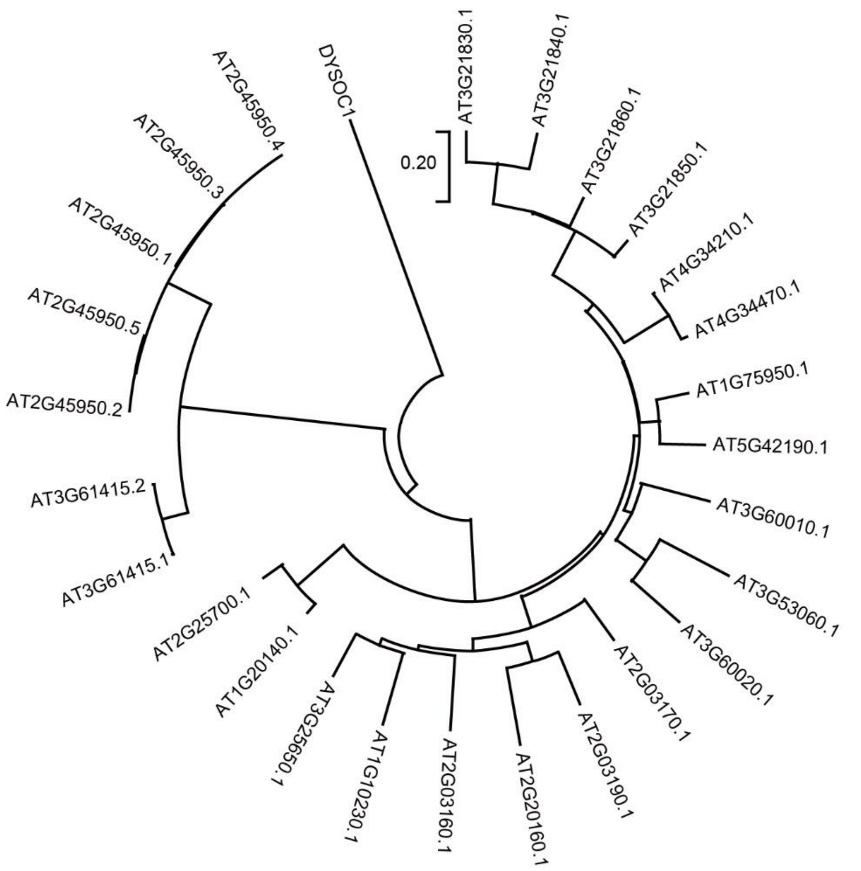

Fig. S25. The phylogenic tree analysis of DYSOC1 and Skp1-like proteins in *Arabidopsis*.

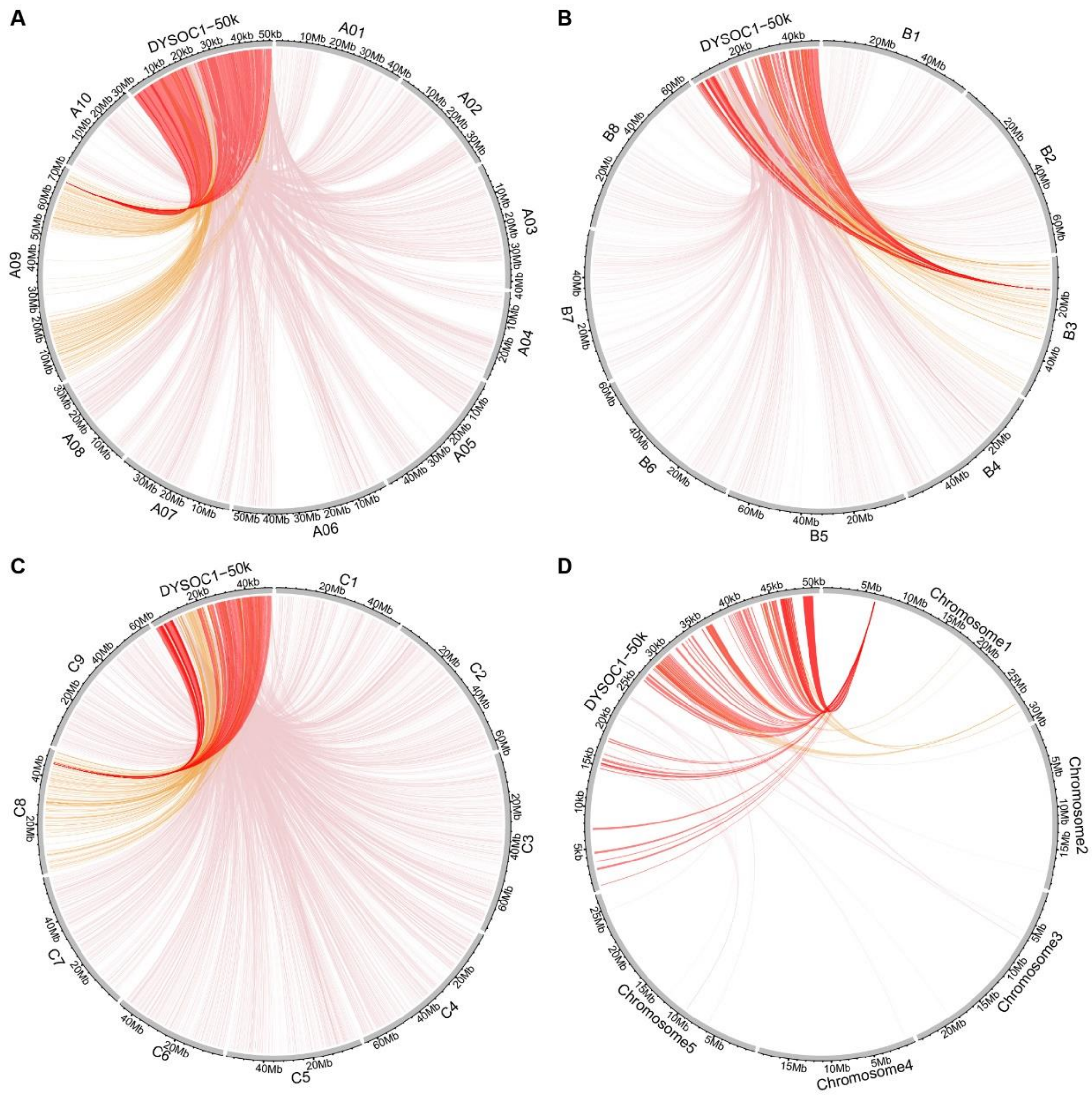

Fig. S26. The micro-synteny analysis of *DYSOC1* and its 25 kb flanks in different species.

The micro-synteny analysis of *DYSOC1* and its 25 kb flanks in *B. rapa* (AA) **(A)**, *B. nigra* (BB) **(B)**, *B. oleracea* (CC) **(C)**, and *A. thaliana* **(D)** genome. The red lines represent the homology sequence of the query segment on the syntenic region. The orange lines represent the homology sequence of the query segment on the ref chromosome that contains the syntenic region. Pink lines represent other homologous sequences.

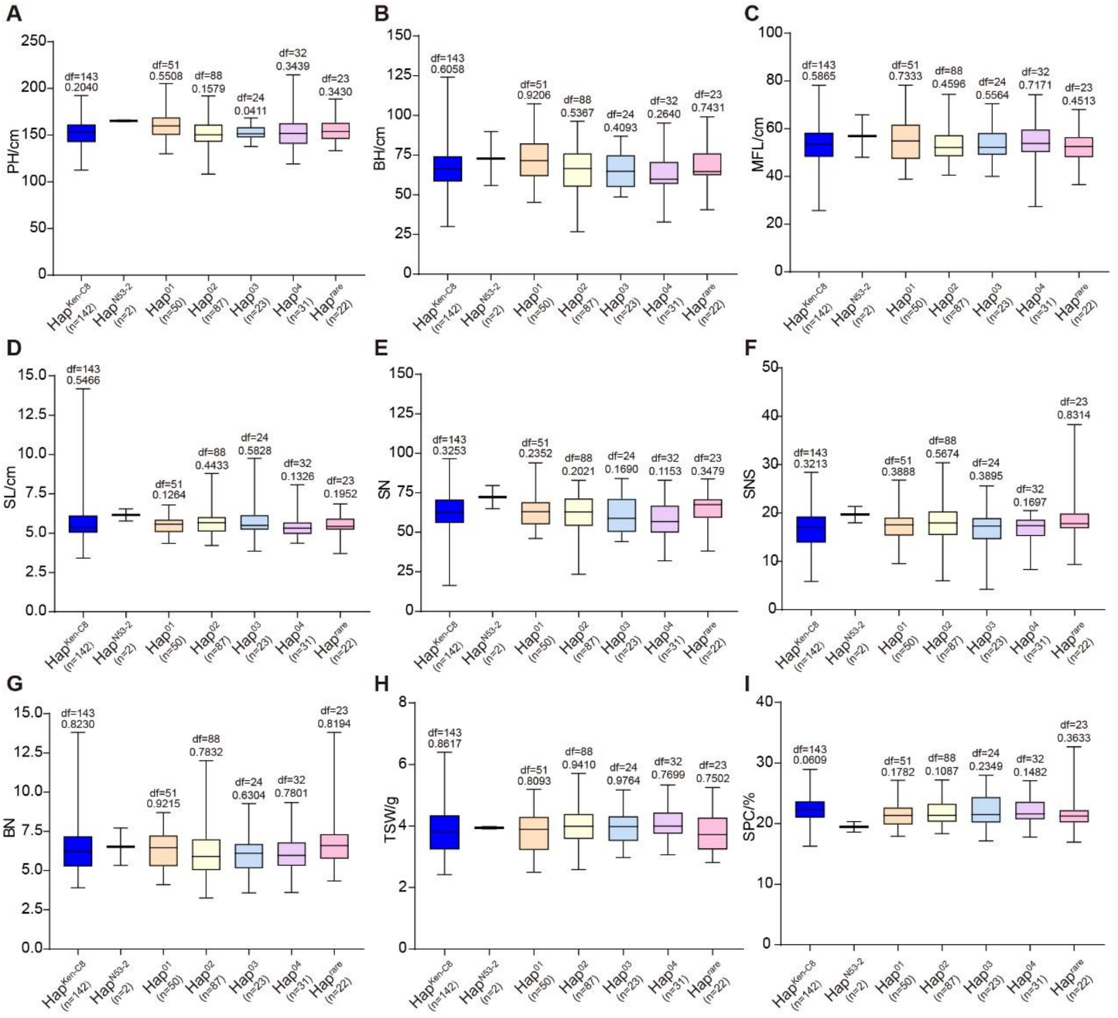

Fig. S27. The plant architecture of different haplotypes.

The PH **(A)**, BH **(B)**, MFL **(C)**, SL **(D)**, SN **(E)**, SNS (**F**), BN **(G)**, TSW **(H)**, and SPC **(I)** of different *BnaA09g44670D* haplotypes in the *B. napus* natural population. The error bars represent the Min and Max values of each haplotype, and the upper, middle, and lower parallel lines represent the first, second, and third quartiles of each haplotype, respectively. The df and *p*-values are annotated above histograms (compared with $Hap^{N53\text{-}2}$, two-tailed one-way ANOVA). In addition, the number of lines of each haplotype is annotated behind the x-axis. For each line, two replications were cultivated, and for each replication, five healthy individuals were randomly selected for phenotype measurement, and the average value was adopted.

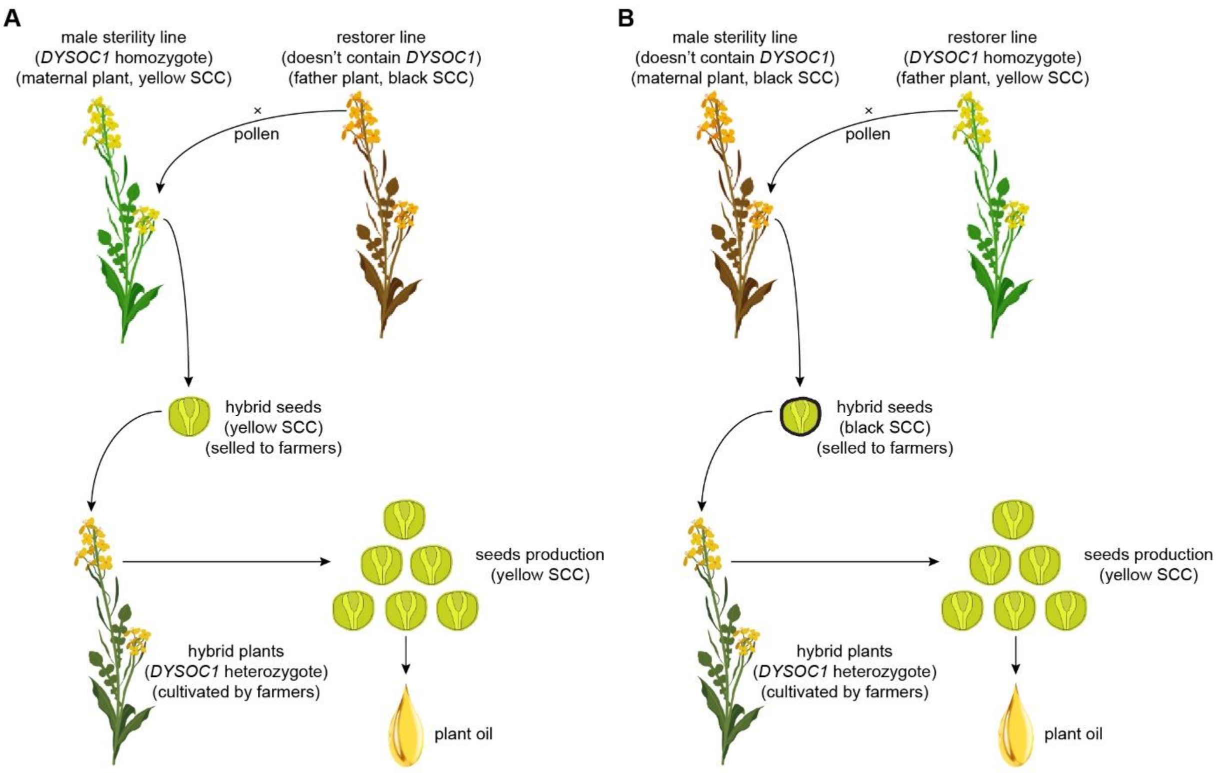

Fig. S28. The schematic diagram of breeding strategies incorporating *DYSOC1* in *B. napus*.

The first strategy can produce yellow SCC hybrid seeds **(A)**; while the second strategy can produce black SCC hybrid seeds **(B)**. Both types of hybrid seeds could be sold to farmers for yellow SCC seeds production, which were subsequently processed for plant oil.

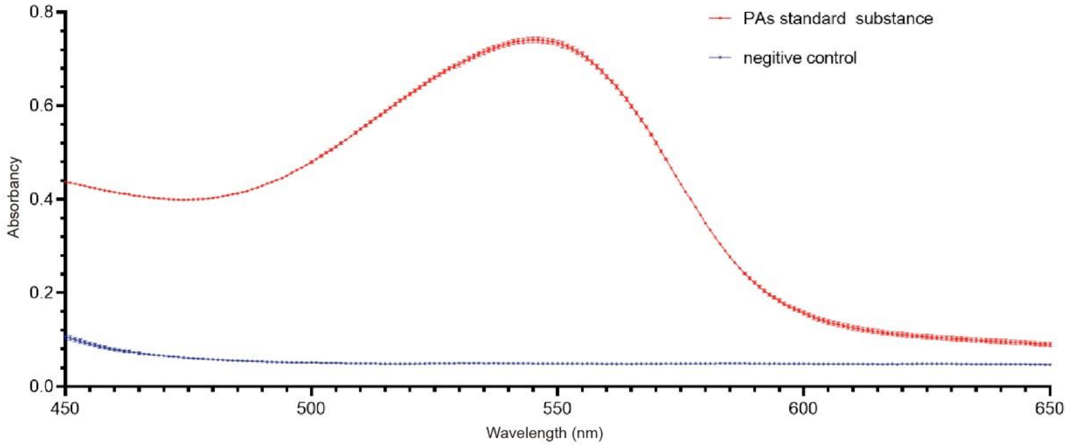

Fig. S29. The absorbancy of the PA extraction system and the negative control.

The maximum absorption peak was observed under 545 nm.

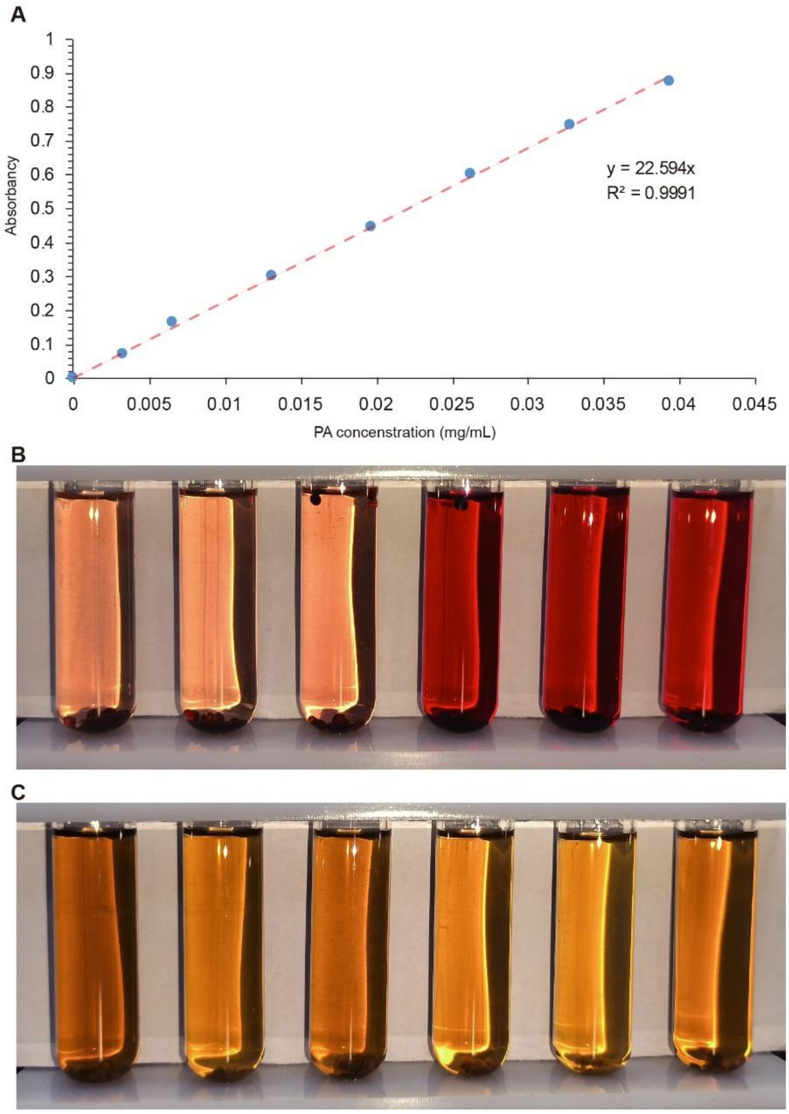

Fig. S30. The PA extraction of *B. napus* seeds.

(A) The standard curve for PA measurement. **(B)** The PA extraction of J9709^{DYSOC1} (left three) and J9709^{WT} (right three) seed coats. **(C)** The PA extraction of J9709^{DYSOC1} (left three) and J9709^{WT} (right three) embryos.

Data S1. (separate file)

Table S1. The details of the RNA-seq of each DH line and the two parents.

Table S2. The genetic linkage map constructed in the present study.

Table S3. The details of all eQTLs.

Table S4. The range of each *trans*-eQTL hotspot.

Table S5. The *trans*-eQTLs within each eQTL hotspot.

Table S6. The GO and KEGG enrichment analysis of *trans*-eQTL hotspot 1.

Table S7. The GO and KEGG enrichment analysis of *trans*-eQTL hotspot 2.

Table S8. The GO and KEGG enrichment analysis of *trans*-eQTL hotspot 3.

Table S9. The GO and KEGG enrichment analysis of *trans*-eQTL hotspot 4.

Table S10. The GO and KEGG enrichment analysis of *trans*-eQTL hotspot 5.

Table S11. The GO and KEGG enrichment analysis of *trans*-eQTL hotspot 6.

Table S12. The GO and KEGG enrichment analysis of *trans*-eQTL hotspot 7.

Table S13. The GO and KEGG enrichment analysis of *trans*-eQTL hotspot 8.

Table S14. The GO and KEGG enrichment analysis of *trans*-eQTL hotspot 9.

Table S15. The GO and KEGG enrichment analysis of *trans*-eQTL hotspot 10.

Table S16. The GO and KEGG enrichment analysis of *trans*-eQTL hotspot 11.

Table S17. The GO and KEGG enrichment analysis of *trans*-eQTL hotspot 12.

Table S18. The GO and KEGG enrichment analysis of *trans*-eQTL hotspot 13.

Table S19. The GO and KEGG enrichment analysis of *trans*-eQTL hotspot 14.

Table S20. The GO and KEGG enrichment analysis of *trans*-eQTL hotspot 15.

Table S21. The GO and KEGG enrichment analysis of *trans*-eQTL hotspot 16.

Table S22. The GO and KEGG enrichment analysis of *trans*-eQTL hotspot 17.

Table S23. The GO and KEGG enrichment analysis of *trans*-eQTL hotspot 18.

Table S24. The GO and KEGG enrichment analysis of *trans*-eQTL hotspot 19.

Table S25. The GO and KEGG enrichment analysis of *trans*-eQTL hotspot 20.

Table S26. The GO and KEGG enrichment analysis of *trans*-eQTL hotspot 21.

Table S27. The GO and KEGG enrichment analysis of *trans*-eQTL hotspot 22.

Table S28. The GO and KEGG enrichment analysis of *trans*-eQTL hotspot 23.

Table S29. The GO and KEGG enrichment analysis of *trans*-eQTL hotspot 24.

Table S30. The GO and KEGG enrichment analysis of *trans*-eQTL hotspot 25.

Table S31. The details of all QTLs that detected in the BC_5F_2 NIL population.

Table S32. Annotated genes within *uqA9-9*.

Table S33. Genes that are highly significantly ($p < 0.01$) co-related to SOC in the WGCNA analysis in ten micro-environments.

Table S34. Genes that are highly significantly ($p < 0.01$) co-related to SCC in the WGCNA analysis in 6 micro-environments.

Table S35. Genes that are consistently highly significantly co-related to SOC or SCC in the WGCNA analysis in all inspected micro-environments.

Table S36. The amino acid sequence of all Skp1-like proteins in *Arabidopsis* and their sequence identities with DYSOC1.

Table S37. The *DYSOC1* homologous sequence in *B.rapa*, *B. nigra*, and *B. oleracea* genome together with their 2 kb promoter.

Table S38. The sequence of $Hap^{Ken\text{-}C8}$, $Hap^{N53\text{-}2}$, and $Hap^{01\text{-}04}$.

Table S39. The primers used in this study.

Table S40. The details of the RNA-seq analysis of NIL materials.

Table S41. The details of the RNA-seq analysis of J9709 and N53-2 transgenic materials.
